# Toward Objective Anal Cancer Screening: From Swab‐Based Genome‐Wide Methylation Marker Discovery to Novel Test Development

**DOI:** 10.1002/ijc.70708

**Published:** 2026-08-27

**Authors:** Kirsten Rozemeijer, Jürgen Claesen, Fernando Dias Gonçalves Lima, Albertus T. Hesselink, Phoebe Klingers, Timo ter Braak, Yara van den Burgt, Chris J. L. M. Meijer, Jan M. Prins, Henry J. C. de Vries, Mark A. van de Wiel, Renske D. M. Steenbergen

**Affiliations:** ^1^ Department of Pathology Amsterdam UMC, location Vrije Universiteit Amsterdam Amsterdam the Netherlands; ^2^ Cancer Center Amsterdam, Imaging and Biomarkers Amsterdam the Netherlands; ^3^ Department of Epidemiology and Data Science Amsterdam UMC, location Vrije Universiteit Amsterdam Amsterdam the Netherlands; ^4^ Amsterdam Public Health Amsterdam the Netherlands; ^5^ Department of Dermatology University of Amsterdam, Amsterdam UMC Amsterdam the Netherlands; ^6^ Self‐Screen B.V. Amsterdam the Netherlands; ^7^ Department of Internal Medicine University of Amsterdam, Amsterdam UMC Amsterdam the Netherlands; ^8^ Center for Sexual Health, Department of Infectious Diseases Public Health Service Amsterdam Amsterdam the Netherlands; ^9^ Amsterdam Institute for Global Health and Development Amsterdam the Netherlands; ^10^ Amsterdam Institute for Infection and Immunity, Infectious Diseases Amsterdam the Netherlands

**Keywords:** anal cancer, biomarker, cancer screening, DNA methylation, genome‐wide profiling study

## Abstract

Anal high‐grade squamous intraepithelial lesions (HSIL:AIN2/3) are precursors to anal cancer, and early detection and treatment through screening is crucial for effective cancer prevention. Current guidelines recommend anal swab‐based screening and highlight the need for objective biomarkers to improve risk stratification. While DNA methylation markers have shown strong potential in anal biopsies, their performance in anal swabs is reduced, likely due to sample‐type dependency. This study aimed to identify a DNA methylation classifier specifically derived from anal swabs of men who have sex with men living with HIV (MSMLWH) to enhance diagnostic accuracy. Genome‐wide methylation profiling was performed on anal swabs from 36 HSIL:AIN3 cases, 34 controls (<low‐grade squamous intraepithelial lesions [<LSIL]), and 7 anal cancer patients using the 850 K Infinium MethylationEPIC BeadChip (Illumina). Candidate CpG sites were selected via logistic regression with adaptive multi‐group ridge penalties using co‐data and an empirical Bayes approach (ecpc). The top candidate markers were validated using quantitative methylation‐specific PCR (qMSP) on an independent series of 204 swabs to identify an optimal methylation classifier. Ecpc analysis identified 50 CpG sites with a combined area‐under‐the‐curve (AUC) of 0.80. All 10 candidate markers evaluated by qMSP showed significant increases in methylation levels associated with disease severity (*p* < 0.001). From these, a 3‐gene methylation classifier (*NOVA1*; *TBXT*; *ZNF788*) was identified, demonstrating excellent performance with an AUC of 0.80 (95% confidence interval: 0.72–0.88) in distinguishing HSIL:AIN3+ from ≤LSIL. This study identified a robust 3‐gene methylation classifier with strong potential for detecting HSIL:AIN3 and anal cancer when applied to anal swabs from MSMLWH.

## What's New?

High‐grade squamous intraepithelial lesions (HSIL) can evolve into anal cancer over time. Recommended swab‐based screening and anal cytology to detect HSIL, however, have limited accuracy, creating a need for objective biomarkers tailored to this sample type. Here, the authors performed genome‐wide DNA methylation profiling of anal swabs from men who have sex with men living with HIV. Candidate markers exhibited increasing methylation with disease severity, leading to the identification of a three‐gene classifier that accurately distinguished HSIL and anal cancer from lower‐grade lesions. The classifier successfully identified all anal cancer cases, supporting further evaluation as a noninvasive screening approach.

## Graphical abstract

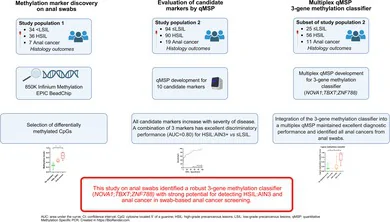

**Abbreviations**
*ACTB*
: β‐actin
AIN: anal intraepithelial neoplasia
AUC: area under the curve
CI: confidence interval
CpG: cytosine located 5′ of a guanine
Cq: quantification cycle
FFPE: formalin‐fixed paraffin‐embedded
H&E: hematoxylin and eosin
HPV: human papillomavirus
HPV16^−^ HSIL: Screening participant with a HSIL diagnoses and no HPV16 infection
HPV16^+^ HSIL: Screening participant with a HSIL diagnoses and HPV16 infection
HRA: high‐resolution anoscopy
hrHPV: high‐risk human papillomavirus
HSIL: high‐grade anal squamous intraepithelial lesion
HSIL:AIN2: high‐grade anal squamous intraepithelial lesion grade 2
HSIL:AIN3: high‐grade anal squamous intraepithelial lesion grade 3
IANS: International Anal Neoplasia Society
LAST: Lower Anogenital Squamous Terminology
LSIL: low‐grade squamous intraepithelial lesion
M+HSIL: HSIL biopsy positive for methylation
M−HSIL: HSIL biopsy negative for methylation
MLWH: men living with HIV
MSMLWH: men who have sex with men living with HIV
ns: nonsignificant
qMSP: quantitative methylation‐specific PCR
ROC: receiver operating characteristics
SCC: squamous cell carcinoma
TWLWH: transgender women living with HIV

## 1 Introduction

The incidence of anal cancer is rising, with men who have sex with men and live with HIV (MSMLWH) at the highest risk [1, 2]. Anal cancer is preceded by high‐grade squamous intraepithelial lesions (HSIL; HSIL:AIN2/3), primarily caused by high‐risk human papillomavirus (hrHPV), predominantly Type 16 [3]. Screening and treatment of HSIL reduces anal cancer risk, enables earlier cancer detection, and improves survival [4, 5].

High‐resolution anoscopy (HRA) with biopsies taken of suspicious lesions remains the diagnostic gold standard for HSIL. However, it is costly, technically demanding, burdensome for patients, and capacity is limited. Consequently, the International Anal Neoplasia Society (IANS) consensus guidelines, followed by several national guidelines, recommend swab‐based screening, including cytology and high‐risk (hr)HPV testing, although both have suboptimal sensitivity and/or specificity [6, 7, 8]. Recent studies and expert opinions have highlighted DNA methylation testing of anal swabs as a promising tool to improve diagnostic accuracy [9, 10, 11, 12, 13, 14, 15, 16, 17].

DNA methylation, an epigenetic modification, can silence tumor suppressor genes, thereby contributing to carcinogenesis [18]. Previous studies on methylation testing of anal biopsies have built upon findings from cervical cancer research, demonstrating that increased methylation of markers originally identified in cervical studies correlates with the severity of anal disease. Specifically, methylation tests combining the markers *ASCL1/SST/ZNF582* and *ASCL1/ZNF582* have shown high accuracy in detecting HSIL lesions and successfully identified all anal squamous cell carcinoma (SCCs) [19, 20, 21]. Furthermore, HSIL lesions exhibited heterogeneous methylation patterns: one subset showed low methylation, while the other displayed a “cancer‐like” methylation pattern characterized by elevated methylation levels. This latter group is believed to represent HSIL lesions with an increased risk of progression to cancer, justifying immediate treatment. Supporting this, longitudinal analyses of HSIL biopsies from patients who during follow‐up developed cancer consistently revealed high scores for the *ASCL1/(SST)/ZNF582* methylation tests [19, 20].

In continuation of these studies, we recently demonstrated the feasibility of applying the same methylation markers to anal swabs rather than biopsies, highlighting their potential for noninvasive HSIL detection [11]. However, performance on swabs was reduced compared to biopsies, likely reflecting sample‐type dependency of methylation marker efficacy. Given this, methylation tests optimized for anal biopsies may not directly translate to anal swabs, underscoring the need to specifically develop methylation classifiers for application on anal swabs. Accordingly, this study was initiated to develop a methylation classifier through genome‐wide methylation profiling of anal swabs collected from men who have sex with men and living with HIV (MSMLWH).

## 2 Material and Methods

### 2.1 Study Design and Participants

Study participants were enrolled via two routes, as previously described [11].

1. Anal cancer screening clinic, Amsterdam UMC: MSMLWH, transgender women living with HIV (TWLWH) aged ≥ 35 years, and individuals with anal symptoms were screened for anal HSIL. Screening consisted of digital anal rectal examination, an anal swab for cytological assessment, and complete visual inspection by HRA with intra‐anal biopsies taken from suspicious lesions according to previous IANS guidelines [22]. Screening participants were invited to join the biobank study starting January 2018, with data on age, sex, gender, and HIV status collected from electronic patient records.
2. Radiotherapy department, Amsterdam UMC: Patients with histologically confirmed intra‐anal SCC were enrolled from January 2018, with anal swabs collected during follow‐up visits, before treatment initiation.

From these study participants, two independent sample sets were established for analysis (Figure S1). To minimize misclassification, all anal swabs were obtained from participants who underwent technically satisfactory HRA and successful biopsy sampling of all lesions identified during HRA.

#### 2.1.1 Set 1: Series for Genome‐Wide Methylation Profiling

The case–control series included anal swabs from 40 men living with HIV (MLWH) diagnosed with HSIL:AIN3 (cases) and 39 MLWH without dysplasia (controls). Additionally, swabs from seven patients with SCC were included for the selection of discriminative CpG sites.

#### 2.1.2 Set 2: Series for Candidate Marker Evaluation and Selection

The independent cross‐sectional series consisted of 185 anal swabs from MLWH (69 no dysplasia, 26 low‐grade squamous intraepithelial lesions [LSIL], 90 HSIL) and 19 swabs from patients with SCC. A subset of 93 samples with sufficient DNA quantity was used to evaluate the 3‐gene methylation classifier.

### 2.2 Procedures and Analysis of Anal Swabs and Biopsies

#### 2.2.1 Collection of Anal Swabs

Anal swabs were collected using a wetted Dacron swab, which was inserted and retracted slowly while rotating and applying lateral pressure. The swab was then transferred to a vial containing 20 mL of ThinPrep medium (Hologic, Bedford, MA, USA), vigorously swirled to release the material for DNA isolation.

#### 2.2.2 Collection of Anal Biopsies and Histopathological Review

Formalin‐fixed and paraffin‐embedded (FFPE) anal biopsies were sectioned using the sandwich method. The first and last sections were stained with hematoxylin and eosin (H&E) and examined by pathologists. LSIL (AIN1) and HSIL (HSIL:AIN2/3) diagnoses were made according to the Lower Anogenital Squamous Terminology (LAST) criteria, using p16 as indicated [23]. In‐between sections were collected for DNA isolation.

#### 2.2.3 DNA Isolation

DNA from anal swabs was isolated using the NucleoMag 96 DNA isolation kit (Macherey‐Nagel, Düren, Germany) and the Microlab Star robotic system (Hamilton, Gräfelfing, Germany). DNA from biopsy tissue sections was isolated using the QIAamp DNA FFPE tissue kit (Qiagen, Hilden, Germany) and eluted with the easyMAG 3 elution buffer (bioMérieux, Boxtel, the Netherlands).

#### 2.2.4 hrHPV Status

Anal swabs were tested for hrHPV as described previously using the GP5+/6+‐PCR EIA followed by Luminex typing or the QIAscreen HPV PCR Test (QIAgen, Venlo The Netherlands; a rebrand of the HPV‐Risk assay) [20, 24]. Both tests are clinically validated for cervical screening purposes according to the international guidelines for HPV test requirements and perform equally [25, 26].

### 2.3 Genome‐Wide Methylation Profiling

Genome‐wide DNA methylation profiling was performed on 500 ng of DNA extracted from anal swabs using the Infinium Human Methylation 850 k BeadChip (Illumina, San Diego, CA, USA) at the Core Facility Genomics of Amsterdam UMC [27].

#### 2.3.1 Filtering of CpG Sites

CpG sites were excluded from the analysis if they (a) had low detection values (i.e., fluorescence is not significantly different from noise, *p >* 0.01) for more than 10% of the samples, (b) had low bead numbers (< 3 in at least 10% of samples), (c) were vulnerable to cross‐hybridization, (e) were defined as problematic by Zhou et al. [28], (f) were SNP's [28], (g) were located on allosomes, (h) were not related to a gene, (i) were not related to a CpG‐island, CpG‐shore, CpG‐shelf, (j) were not located in a gene or a CpG island, and/or (k) were located on the sex chromosomes.

#### 2.3.2 Transformation and Normalization of CpG Sites

The intensity values of the remaining CpG sites were transformed to beta‐values (ratio of intensities between methylated and unmethylated CpGs) [29] and subsequently normalized using the functional normalization procedure as implemented in the *Mefill* R package [https://github.com/perishky/meffil].

#### 2.3.3 Sample Quality Control

The quality of the samples was checked according to the control metrics specified by Illumina [30]. Samples that did not meet the control metrics and those with low detection values (i.e., fluorescence was not significantly different from noise, *p >* 0.01) for more than 90% of the remaining CpG sites were excluded.

### 2.4 DNA Methylation Testing by qMSP

Methylation analysis was performed on isolated DNA of both anal swabs and biopsies, which was bisulfite‐converted using the EZ DNA Methylation kit (Zymo Research, Orange, CA, USA) following the manufacturer's instructions. EpiTect MethyLite Master Mix (Qiagen, Hilden, Germany) was employed along with fluorescent dye‐labeled probes, utilizing 50–100 ng of bisulfite‐converted DNA as input and 100–300 nM of each primer. These specific primers amplified the methylated bisulfite converted DNA. The methylation assays were conducted on the ViiA 7 Real‐Time PCR System (Applied Biosystems, Foster City, CA, USA) with a double‐stranded DNA‐based calibrator used as an internal quality control. As a negative control, H2O was used, and β‐actin (*ACTB*) served as the reference gene. Cycle threshold values were determined at fixed fluorescence thresholds and an acceptable cycle threshold (Cq) of < 32 for *ACTB* indicated the presence of sufficient DNA, good DNA quality, and successful bisulfite conversion. Methylation values were normalized to *ACTB* and the calibrator using the comparative Cq method (2–ΔΔCq × 100), resulting in ΔΔCq ratios [22, 23].

#### 2.4.1 Testing of the Anal Swabs

qMSPs for 10 candidate markers (see Section 2.5.2) were developed as previously described [31]. Two to four markers, including the reference gene *ACTB*, were combined in a multiplex qMSP. The *ACTB* reference gene was used for quantification and quality control. Multiplex qMSP1 comprised *ACTB*, *CTNND2*, *WDR17*, and *ZNF788*. Multiplex qMSP2 consisted of *ACTB*, *NPY5R*, *TBXT*, and *ZNF781*. Multiplex qMSP3 comprised *ACTB*, *GALR1*, *NID2*, and *SLC46A3*. Multiplex qMSP4 consisted of *ACTB* and *NOVA1*.

A multiplex qMSP for the best performing 3‐gene methylation classifier (see Section 2.5.4) (*NOVA1*, *TBXT*, and *ZNF788*) and the reference gene *ACTB* was designed and analytically validated by Self‐screen BV.

#### 2.4.2 Testing of the Anal Biopsies

Anal HSIL biopsies of Set 1 were analyzed with the validated methylation marker panel *ASCL1/SST/ZNF582*, as described previously [19, 20]. Methylation positivity was determined based on a previously defined threshold of optimal sensitivity and specificity (Youden J index). At this threshold, all HSIL lesions that progressed to cancer over time in a longitudinal series, as well as all cancers, tested positive for methylation [20].

Anal HSIL biopsies of Set 2 were analyzed with the *ASCL1/ZNF582* methylation test, PreCursor‐M AnoGYN (Self‐screen, Amsterdam, the Netherlands), as described previously [19]. The threshold was set at 0.5, a level at which all HSIL lesions that progressed to cancer over time in a longitudinal series, as well as all cancers, tested positive for methylation.

Methylation test results from the *ASCL1/SST/ZNF582* and *ASCL1/ZNF582* methylation tests were used as proxies for increased risk of progression to anal cancer, as established in our previous studies [19, 20]. Based on these results, HSIL lesions were categorized into methylation‐positive biopsies (M^+^ HSIL; M^+^ HSIL:AIN3), indicative of higher cancer risk, and methylation‐negative biopsies (M^−^ HSIL; M^−^ HSIL:AIN3), associated with lower cancer risk.

### 2.5 Data Analysis and Statistics

#### 2.5.1 Selection of CpG Sites

After pre‐processing the DNA methylation data, we selected differentially methylated CpG sites by fitting a logistic regression model penalized with group‐adaptive, complementary data (co‐data) learned, ridge penalties.

Previously published methylation profiling data of anal tissue samples were used as co‐data. In this previous study, 274 differentially methylated CpG sites were identified as differentially methylated when comparing normal to cancerous anal tissues [32]. Incorporation of this co‐data guided marker selection by enhancing the identification of valuable markers from impure samples, such as anal swabs [27]. The use of co‐data helps to narrow the search space and directs attention toward the most informative regions.

In high‐dimensional settings, ridge and lasso regression models are commonly applied when selecting features or for predictions. These models penalize the (down‐weigh) regression coefficients. By adequately estimating penalties or weights from informative co‐data, the CpG site selection and prediction for high‐dimensional data can be improved. We estimated group‐adaptive weights using a moment‐based empirical Bayes approach [33]. Group‐adaptive weights allow the selection of groups of CpG sites that share the same penalty parameterized by a group‐specific hyper‐parameter. These hyper‐parameters are estimated from the data, showing how informative the co‐data are and how important each CpG group is for the feature selection problem. Important CpG sites will have no or a small penalty, whereas unimportant CpG sites will have a large penalty significantly down‐weighing the regression coefficients.

The performance of the logistic regression model was quantified using the Brier‐score and area under the ROC‐curve (AUC) value, both obtained by 10‐fold cross‐validation. Additionally, prediction probabilities for HSIL:AIN3 were calculated using a fitted regression model. The model incorporating CpG sites with the highest discriminative power, as determined by the maximum AUC, was selected, and a heatmap was generated to visualize these CpG sites.

#### 2.5.2 Selection of Candidate Markers

To identify candidate markers with the highest potential, we compared methylation levels of individual CpG sites selected by the logistic regression model. Differences in DNA methylation levels between disease categories were evaluated using the Kruskal–Wallis omnibus test, followed by post hoc pairwise Wilcoxon‐Mann–Whitney *U*‐tests with Bonferroni correction for multiple testing. To distinguish between HSIL:AIN3 lesions with low versus high cancer risk, we classified HSIL:AIN3 based on the methylation status of their biopsies determined by the *ASCL1/SST/ZNF582* 3‐gene methylation classifier: M^−^ HSIL:AIN3 indicated lower risk, while M^+^ HSIL:AIN3 indicated higher risk. The following criteria were applied: (a) methylation levels should progressively increase from no dysplasia through M^−^ HSIL:AIN3 and M^+^ HSIL:AIN3 to SCC; (b) a *p* value of ≤ 0.0005, when comparing methylation levels between SCC and no dysplasia; and (c) a *p* value of ≤ 0.05, when comparing methylation levels between M^+^ HSIL:AIN3 and no dysplasia. If multiple CpG sites from a single gene were selected, the most discriminative CpG site was retained.

#### 2.5.3 Analysis of qMSP Results

To visualize the methylation levels in anal swabs across disease categories, boxplots were generated using square‐root transformed ΔΔCq ratios. Differences in DNA methylation between disease categories were assessed with the Kruskal–Wallis omnibus test, followed by post hoc pairwise Wilcoxon‐Mann–Whitney U‐tests with Bonferroni correction for multiple testing. The primary outcomes focused on differences among no dysplasia, LSIL, HSIL, and SCC. Secondary outcomes included risk stratification of HSIL lesions. Hereto, HSIL lesions were classified according to their risk of progression to anal cancer based on their methylation status (as determined by the *ASCL1/ZNF582* methylation test on HSIL biopsy samples; see Section 2.4.2) and HPV16 infection status (as determined by hrHPV testing of anal swabs). High‐risk lesions included methylation‐positive biopsies (M^+^ HSIL) or a HPV16‐positive status (HPV16^+^ HSIL), while low‐risk lesions included methylation‐negative biopsies (M^−^ HSIL) or a HPV16‐negative status (HPV16^−^ HSIL). Accordingly, differences were examined between no dysplasia, LSIL, M^−^ HSIL, M^+^ HSIL, and SCC, as well as no dysplasia, LSIL, HPV16^−^ HSIL, HPV16^+^ HSIL, and SCC.

#### 2.5.4 Building of the Methylation Classifier

We used a proportional odds cumulative ordinal regression model with an elastic net penalty to determine a 3‐ and 5‐gene methylation classifier [34, 35]. Considering disease status as an ordered categorical response variable with four levels (≤LSIL, HSIL:AIN2, HSIL:AIN3, SCC), we evaluated the regression model's prediction accuracy for both individual categories (≤LSIL vs. HSIL:AIN2 vs. HSIL:AIN3 vs. SCC) and for combined groupings, such as ≤LSIL versus HSIL:AIN3 and SCC. We compared the prediction accuracies of the ordinal regression model with those of the corresponding random forest model [36]. When combining the four categories to two outcomes, we compared the performance against a logistic regression model with an elastic net penalty. Using this approach, the classifier with the highest AUC was selected.

#### 2.5.5 Performance Analysis of the Methylation Classifier

For the methylation classifier, logistic regression analyses were performed to assess the risk of HSIL:AIN3+ (HSIL:AIN3 or anal SCC) for each sample, expressed as a predicted probability or classifier score ranging from 0 to 1. Cases were defined as HSIL:AIN3+, and controls as ≤LSIL. These predicted probabilities or classifier scores were used to generate a receiver operating characteristic (ROC) curve, and classifier performance was evaluated using the AUC. AUC values were interpreted according to the criteria established by Hosmer and Lemeshow: 0.7–0.8 indicating acceptable discrimination; 0.8–0.9 excellent discrimination; and ≥ 0.9 indicating outstanding discrimination [37]. Subsequently, detection rates corresponding to predefined thresholds were determined.

#### 2.5.6 Statistical Analyses

Statistical analyses were performed with: SPSS version 28; GraphPad Prism version 10.2.0 for Windows, GraphPad Software, Boston, Massachusetts USA, www.graphpad.com; R Statistical Software (version 4.3.2) in RStudio, using the following R‐packages: *ecpc*, *Mefill, ordinalNet, pROC*, *randomForest*.

## 3 Results

### 3.1 Genome‐Wide Methylation Profiling

Genome‐wide methylation profiling was performed on swabs from 79 screening participants, 40 HSIL:AIN3 and 39 without dysplasia, and swabs from 7 patients with SCC (Set 1). Nine swabs did not pass the sample quality control, resulting in swabs from 70 screening participants and 7 patients with SCC for further analysis (Figure S1). The baseline characteristics are listed in Table 1. Following our selection criteria, 282,378 CpG sites were included in further analysis (Figure S2).

**TABLE 1:** Characteristics of study participants.

	Set 1: Series for genome‐wide methylation profiling	Set 2: Series to evaluate 10 candidate markers	Subset of Set 2: Series to evaluate the 3‐gene classifier multiplex qMSP
*n*	77	203	92
Participant characteristics			
Age in years, median (range)	53 (33–82)	54 (30–76)^a^	53 (31–75)^a^
Sex and gender^b^			
Cisgender man	77 (100.0%)	197 (97.0%)	90 (97.8%)
Cisgender woman	0 (0.0%)	6 (all cancer) (3.0%)	2 (all cancer) (2.2%)
First‐time screeners^c^	24 (31.2%)	28 (13.8%)	18 (19.6%)
Treated in the past for HSIL^c^	34 (44.2%)	107 (52.7%)	42 (45.7%)
HIV status			
Positive	75 (97.4%)	192 (94.6%)	86 (93.5%)
Negative	2 (2.6%)	11 (all cancers) (5.4%)	6 (all cancers) (6.5%)
hrHPV in anal swab			
Positive	57 (74.0%)	134 (66.0%)	72 (78.3%)
Negative	19 (24.7%)	64 (31.5%)	19 (20.7%)
Unknown	1 (1.3%)	5 (2.5%)	1 (1.1%)
Histopathological diagnosis			
No dysplasia	34 (44.2%)	68 (33.5%)	15 (16.3%)
LSIL	0 (0.0%)	26 (12.8%)	10 (10.9%)
HSIL:AIN2	0 (0.0%)	56 (27.6%)	28 (30.4%)
HSIL:AIN3	36 (46.8%)	34 (16.7%)	28 (30.4%)
SCC	7 (9.1%)	19 (9.4%)	11 (12.0%)
HSIL risk stratification according to methylation status of the biopsy, *n* (%) of HSIL^d^			
M^+^ HSIL	16 (44.4%)	39 (43.3%)	23 (41.1%)
M^−^ HSIL	14 (38.9%)	36 (40.0%)	19 (33.9%)
Unknown	6 (16.7%)	15 (16.7%)	14 (25.0%)
HSIL risk stratification according to hrHPV16 status of the swab, *n* (%) of HSIL			
HPV16^+^ HSIL	20 (55.6%)	24 (26.7%)	13 (23.2%)
HV16^−^ HSIL	12 (33.3%)	64 (71.1%)	41 (73.2%)
Unknown	4 (11.1%)	2 (2.2%)	2 (3.6%)

*Note:* Numbers are *n* (%) unless stated otherwise.

Abbreviations: HPV16^−^ HSIL, Screening participant with a HSIL diagnoses and no HPV16 infection (as determined by hrHPV testing of the anal swab); HPV16^+^ HSIL, Screening participant with a HSIL diagnoses and HPV16 infection (as determined by hrHPV testing of the anal swab); HSIL, high‐grade squamous intraepithelial lesions; LSIL, low‐grade squamous intraepithelial lesions; M^−^ HSIL, HSIL biopsy negative for methylation (i.e., determined by the *ASCL1/SST/ZNF582* or *ASCL1/ZNF582* methylation tests); M^+^ HSIL, HSIL biopsy positive for methylation (i.e., determined by the *ASCL1/SST/ZNF582* or *ASCL1/ZNF582* methylation tests); qMSP, quantitative methylation‐specific PCR; SCC, anal squamous cell carcinoma.

^a^ Age is unknown for one the screening participants.

^b^ Cisgender = gender identity corresponds to sex assigned at birth.

^c^ Screening history was unknown for anal cancer patients.

^d^ HSIL lesions were stratified by biopsy methylation status, classified as positive or negative using the *ASCL1/SST/ZNF582* and *ASCL1/ZNF582* methylation tests, both proxies for an increased risk of progression to anal cancer as demonstrated in our previous studies [19, 20, 21].

### 3.2 Selection of Candidate Methylation Markers

Adaptive group‐regularized co‐data learnt ridge regression on the DNA methylation profiles of screening participants with HSIL:AIN3 versus participants with <LSIL (i.e., Set 1) yielded a combination of 50 CpG sites with the highest AUC value (i.e., 0.80). The addition of extra CpGs did not increase the AUC value (Figure S3). The corresponding heatmap is shown in Figure 1.

**FIGURE 1:**
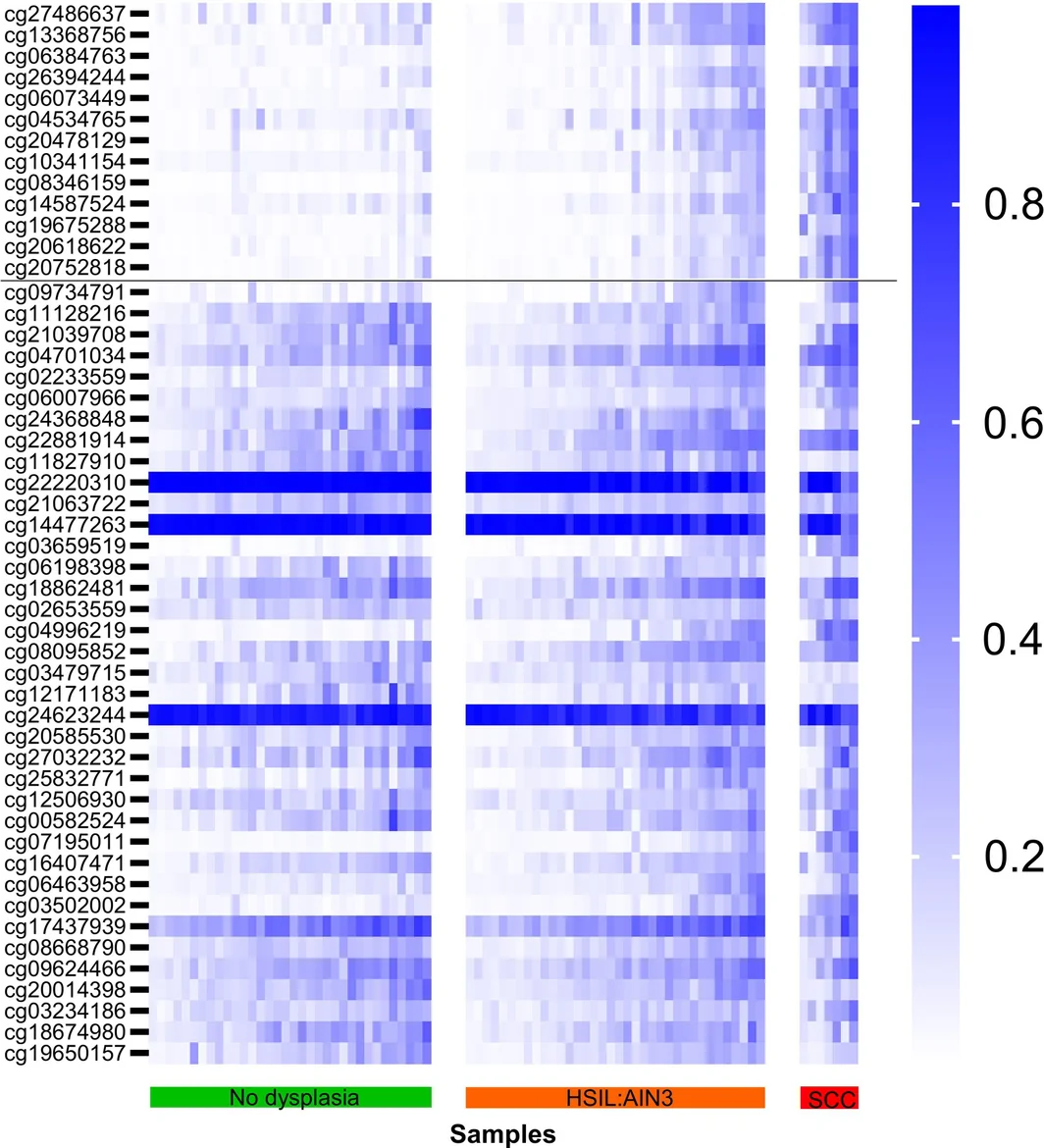
Heatmap of the 50 discriminative CpGs selected by adaptive group‐regularized co‐data learnt ridge regression. DNA methylation levels for each CpG site (indicated by cg‐numbers) are visualized on a color scale from low (light blue) to high (dark blue). Each row represents a CpG site, and each column corresponds to an individual anal swab sample. Samples (columns) were grouped by disease category, as shown by the color bar beneath the heatmap: No dysplasia (green; *n* = 34), HSIL:AIN3 (orange; *n* = 36), and SCC (red; *n* = 7), and ordered by the average methylation values across the 50 CpG sites. The 13 CpG sites above the black horizontal line fulfilled predefined selection criteria: (a) methylation levels increased progressively across disease stages (from no dysplasia to M^−^ HSIL:AIN3, M^+^ HSIL:AIN3, and SCC); (b) methylation levels in SCC differed significantly from those in no dysplasia (*p* ≤ 0.0005); (c) methylation levels in M^+^ HSIL:AIN3 differed significantly from those in no dysplasia (*p* ≤ 0.05). CpG sites (rows) above and below the black line were ranked by importance according to adaptive group‐regularized co‐data‐learned ridge regression. Abbreviations: AIN, Anal intraepithelial lesion; cg, Cytosine located 5′ of a guanine; M^−^ HSIL:AIN3, High‐grade squamous intraepithelial lesions–anal intraepithelial neoplasia grade 3 biopsy negative for methylation (i.e., determined by the *ASCL1/SST/ZNF582* methylation test); M^+^ HSIL:AIN3, High‐grade squamous intraepithelial lesions–anal intraepithelial neoplasia grade 3 biopsy positive for methylation (i.e., determined by the *ASCL1/SST/ZNF582* methylation test); SCC, Anal squamous cell carcinoma.

Thirteen CpG sites, representing ten candidate methylation markers, met the predefined selection criteria. While for *NPY5R* three CpG sites met the criteria, we excluded one CpG site because of its lower discriminative ability in differentiating M^+^ HSIL:AIN3 from no dysplasia compared to the other two selected CpG sites. For *CTNND2*, two additional CpG sites were incorporated based on their proximity to each other. This resulted in the ultimate selection of 14 CpG sites corresponding to 10 candidate methylation markers for qMSP design, allowing for further evaluation using another technique.

### 3.3 Analysis of qMSP Results

#### 3.3.1 Marker Evaluation

The 10 qMSPs designed for the candidate markers were tested on an independent set of 204 anal swabs from screening participants and patients with anal cancer (Set 2). One swab was excluded due to a technical failure, resulting in 203 swabs for analysis (68 no dysplasia, 26 LSIL, 90 HSIL, and 19 patients with SCC) (Table 1, Figure S1). Methylation levels of all 10 markers differed significantly across histological disease categories (*p* < 0.001) (Figure 2). All markers showed significantly higher methylation levels in patients with SCC than in screening participants with HSIL (*p* < 0.001), LSIL (*p* < 0.01), or no dysplasia (*p* < 0.01). For most markers, methylation levels were also significantly elevated in screening participants with HSIL as compared to those without dysplasia. Both the heatmap and boxplots revealed a heterogeneous methylation pattern among swabs of patients with histological HSIL (Figures 1 and 2).

**FIGURE 2:**
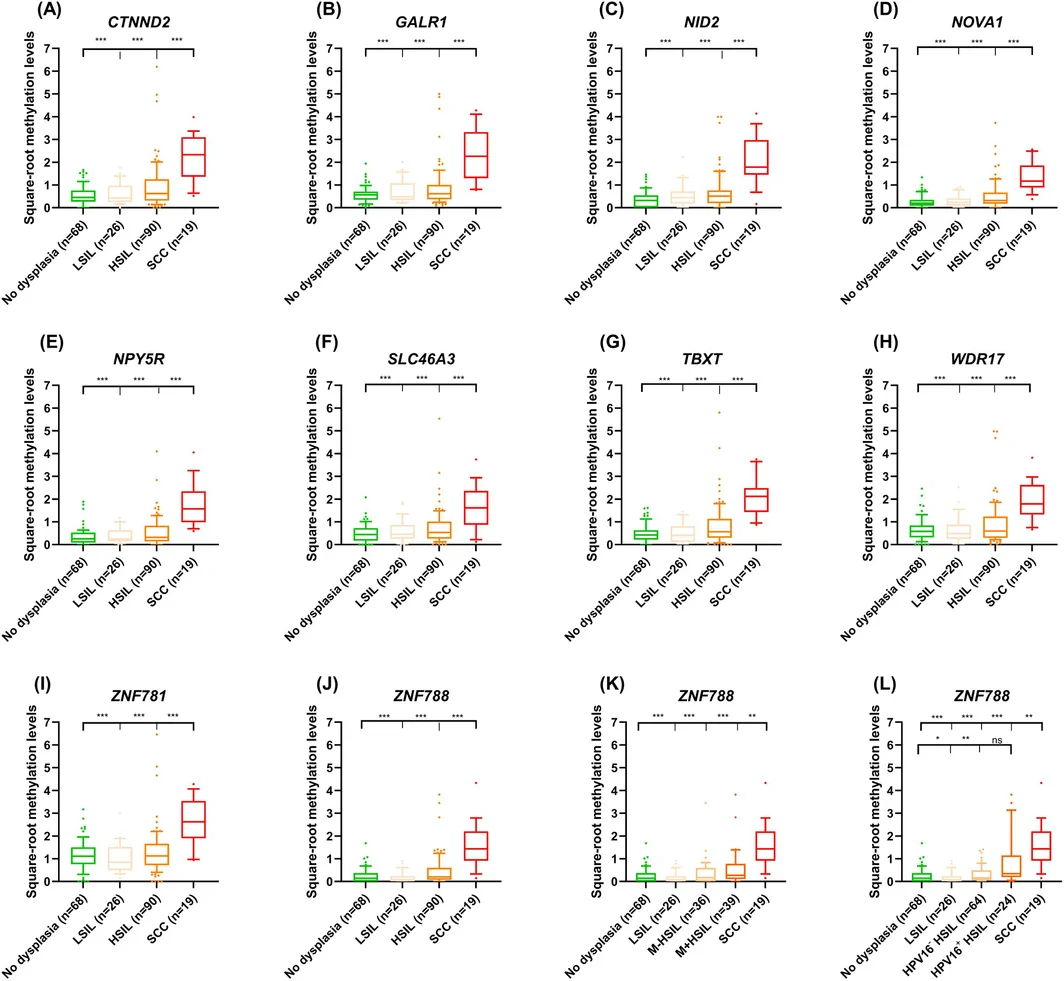
Methylation levels of the 10 candidate markers in anal swabs. DNA methylation levels measured by qMSP and expressed as square‐root transformed ΔΔCq ratios, are plotted against primary histological outcomes (A–J: All markers) and secondary histological outcomes (K and L; ZNF788 only as a representative, see Figures S4 and S5 for all markers). Statistical difference was assessed by the Mann–Whitney *U* test corrected for multiple testing and shown for SCC versus other histological categories and for (L) HPV16^+^ HSIL versus other histological categories. Boxplots display the median (solid line), interquartile range (25th [Q1] to 75th [Q3] percentile), and whiskers indicate the 10th to 90th percentiles. (A–J) Primary outcome: Histological outcomes are categorized as no dysplasia, LSIL, HSIL, and SCC. (K) Secondary outcome: HSIL lesions are further classified based on biopsy methylation status and classified as positive or negative according to the *ASCL1/ZNF582* methylation test, a proxy for increased anal cancer risk as demonstrated in our previous studies [19, 20]. HSIL with biopsies negative for methylation (M^−^ HSIL) represent low‐risk lesions, while HSIL with biopsies positive for methylation (M^+^ HSIL) represent high‐risk lesions. Fifteen swabs from screening participants with HSIL were excluded due to unknown biopsy methylation status. (L) Secondary outcome: HSIL lesions are classified by HPV16 infection status: HPV16‐negative HSIL (HPV16^−^ HSIL) represent low‐risk lesions, while HPV16‐positive HSIL (HPV16^+^ HSIL) represent high‐risk lesions. Two swabs were excluded due to unknown HPV16 status. Statistical differences are also shown for HPV16^+^ HSIL compared to other histological groups. For *NID2 and WDR17*, one datapoint is not shown (NID2: No dysplasia, 13.00; WDR17: No dysplasia, 7.84). *p ≤ 0.05; **p ≤ 0.01; ***p ≤ 0.001. Abbreviations: HPV16^−^ HSIL, Screening participant with a HSIL diagnoses and no HPV16 infection (as determined by hrHPV testing of the anal swab); HPV16^+^ HSIL, Screening participant with a HSIL diagnoses and HPV16 infection (as determined by hrHPV testing of the anal swab); HSIL, High‐grade squamous intraepithelial lesion; LSIL, Low‐grade squamous intraepithelial lesion; M^−^ HSIL, HSIL biopsy negative for methylation (i.e., determined by the *ASCL1/ZNF582* methylation test); M^+^ HSIL, HSIL biopsy positive for methylation (i.e., determined by the *ASCL1/ZNF582* methylation test); ns, non‐significant; SCC, Anal squamous cell carcinoma.

In secondary analyses, methylation‐negative and methylation‐positive HSIL (M^−^ HSIL and M^+^ HSIL) were added as disease categories to classify HSIL lesions by their risk of progression to cancer according to the *ASCL1/ZNF582* methylation test results on anal biopsies. Screening participants with M^+^ HSIL lesions showed significantly higher methylation levels than those with M^−^ HSIL lesions for markers *CTNND2*, *GALR1*, *NID2, NOVA1*, and *NPY5R* after multiple testing correction (*p* < 0.05) (Figure S4). Additionally, HPV16 infection, known to be more oncogenic than most other HPV types, was included as a disease category to further classify HSIL lesions by their risk of progression to anal cancer. Screening participants with HPV16^+^ lesions showed significantly higher methylation levels than those with HPV16^−^ lesions for markers *GALR1*, *NID2, NOVA1*, and *NPY5R* after correction for multiple testing (*p* < 0.05) (Figure S5).

#### 3.3.2 Methylation Classifier Performance

With histological HSIL:AIN3+ as cases and ≤LSIL as controls, the proportional odds cumulative ordinal regression model revealed *NOVA1*, *TBXT*, and *ZNF788* as best performing 3‐gene methylation classifier with an AUC of 0.80 (95% CI: 0.72–0.88) (Figure 3). The optimal 5‐gene methylation classifier consisted of *NOVA1*, *TBXT*, *WDR17*, *ZNF781*, and *ZNF788* and had a similar AUC value of 0.82 (95% CI: 0.75–0.90).

**FIGURE 3:**
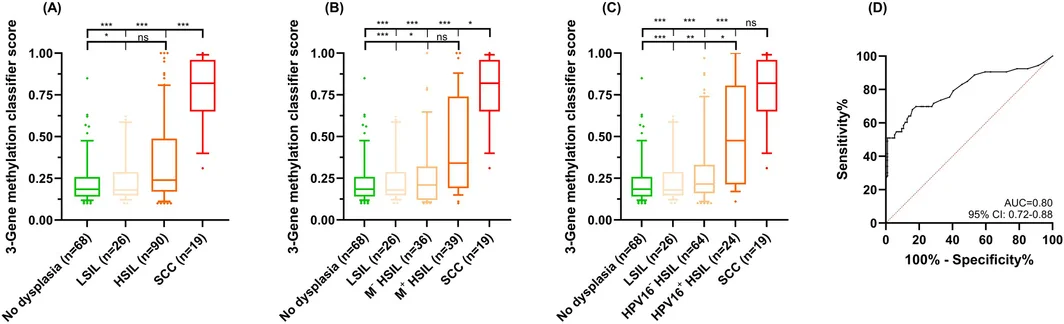
3‐Gene methylation classifier performance: Primary outcome (A), secondary outcomes (B and C), and ROC curve (D). (A–C) The 3‐gene methylation classifier scores are plotted against primary (A) and secondary (B and C) histological outcomes. Statistical difference was assessed by the Mann–Whitney *U* test corrected for multiple testing and shown for SCC versus other histological categories and for (A) HSIL versus other histological categories, (B) M^+^HSIL versus other histological categories, and (C) HPV16^+^ HSIL versus other histological categories. Solid line represents the median, boxplot represents the 25th (Q1) and 75th (Q3) percentile. Whiskers range from the 10th to the 90th percentile. (D) Cases were defined as swabs of screening participants and patients with HSIL:AIN3 or SCC. Controls were defined as swabs of screening participants with ≤LSIL. *p ≤ 0.05; **p ≤ 0.01; ***p ≤ 0.001. Abbreviations: AIN, Anal intraepithelial lesion; AUC, Area under the curve; CI, Confidence interval; HPV16^−^ HSIL, Screening participant with a HSIL diagnoses and no HPV16 infection (as determined by hrHPV testing of the anal swab); HPV16^+^ HSIL, Screening participant with a HSIL diagnoses and HPV16 infection (as determined by hrHPV testing of the anal swab); LSIL, Low‐grade squamous intraepithelial lesion; M^−^ HSIL, HSIL biopsy negative for methylation (i.e., determined by the *ASCL1/ZNF582* methylation test); M^+^ HSIL, HSIL biopsy positive for methylation (i.e., determined by the *ASCL1/ZNF582* methylation test); ns, non‐significant; ROC, Receiver‐operating characteristic curve; SCC, Anal squamous cell carcinoma.

The 3‐gene methylation classifier (*NOVA1*, *TBXT*, and *ZNF788*) demonstrated significantly elevated scores in patients with SCC compared to screening participants with HSIL (*p* < 0.001), LSIL (*p* < 0.001), and no dysplasia (*p* < 0.001), as well as in participants with HSIL versus no dysplasia (*p* < 0.05) (Figure 3). When HSIL cases were stratified by biopsy methylation status according to the *ASCL1/ZNF582* test, as an indication of their cancer risk, M^+^ HSIL lesions tended to have higher 3‐gene methylation classifier scores than M^−^ HSIL lesions. However, this difference was not statistically significant after correction for multiple testing (*p* = 0.08). Stratification by HPV16 infection status revealed significantly increased 3‐gene methylation classifier scores in HPV16^+^ HSIL lesions compared to HPV16^−^ lesions (*p* < 0.05).

The 3‐gene classifier detection rates at thresholds of 0.20, 0.25 and 0.30 are shown in Table 2. Using a threshold of 0.30, all anal cancers were detected. Detection rates for other lesions were 52.9% for HSIL:AIN3, 62.5% for HPV16^+^ HSIL, 56.4% for M^+^ HSIL, 36.7% for overall HSIL, 23.1% for LSIL, and 16.2% for samples without dysplasia. Lowering the threshold to 0.20 increased detection rates across all categories, identifying all anal cancers, 73.5% of HSIL:AIN3, 87.5% of HPV16^+^ HSIL, 69.2% of M^+^ HSIL, 62.2% of HSIL, 46.2% of LSIL, and 41.2% of samples without dysplasia (Table 2). Increasing the threshold to 0.40, 18 out of 19 cancers (94.7%) were detected, while also detecting 47.1% of HSIL:AIN3, 50.0% of HPV16^+^ HSIL, 41.0% of M^+^ HSIL, 26.7% of HSIL, 15.4% of LSIL, and 13.2% of samples without dysplasia.

**TABLE 2:** Detection rates of the 3‐gene methylation classifier.

Classifier score threshold	Primary outcomes					Secondary outcomes as proxy for HSIL lesions with an increased anal cancer risk	
	No dysplasia	LSIL	HSIL	HSIL:AIN3	SCC	M^+^ HSIL	HPV16^+^ HSIL
0.20	28/68 (41.2%)	12/26 (46.2%)	56/90 (62.2%)	25/34 (73.5%)	19/19 (100.0%)	27/39 (69.2%)	21/24 (87.5%)
0.25	18/68 (26.5%)	8/26 (30.8%)	43/90 (47.8%)	18/34 (52.9%)	19/19 (100.0%)	24/39 (61.5%)	18/24 (75.0%)
0.30	11/68 (16.2%)	6/26 (23.1%)	33/90 (36.7%)	18/34 (52.9%)	19/19 (100.0%)	22/39 (56.4%)	15/24 (62.5%)

Abbreviations: HPV16^+^ HSIL, Screening participant with a HSIL diagnosis and HPV16 infection (as determined by hrHPV testing of the anal swab); HSIL, high‐grade squamous intraepithelial lesions; LSIL, low‐grade squamous intraepithelial lesion; M^+^ HSIL, HSIL biopsy positive for methylation (i.e., determined by the *ASCL1/ZNF582* methylation test); SCC, anal squamous cell carcinoma.

#### 3.3.3 Testing of the Classifier Multiplex qMSP

A multiplex qMSP assay incorporating the 3‐gene methylation classifier (*NOVA1*, *TBXT, ZNF788*) along with the reference gene *ACTB* was evaluated on a subset of 92 samples from Set 2. This subset was comprised of 15 samples without dysplasia, 10 with LSIL, 56 with HSIL, and 11 with SCC (Table 1, Figure S1). Both individual gene markers and the combined 3‐gene methylation classifier demonstrated significantly elevated methylation scores in SCC patients compared to screening participants with HSIL (*p* < 0.01), LSIL (*p* < 0.01), and no dysplasia (*p* < 0.001) (Figure 4).

**FIGURE 4:**
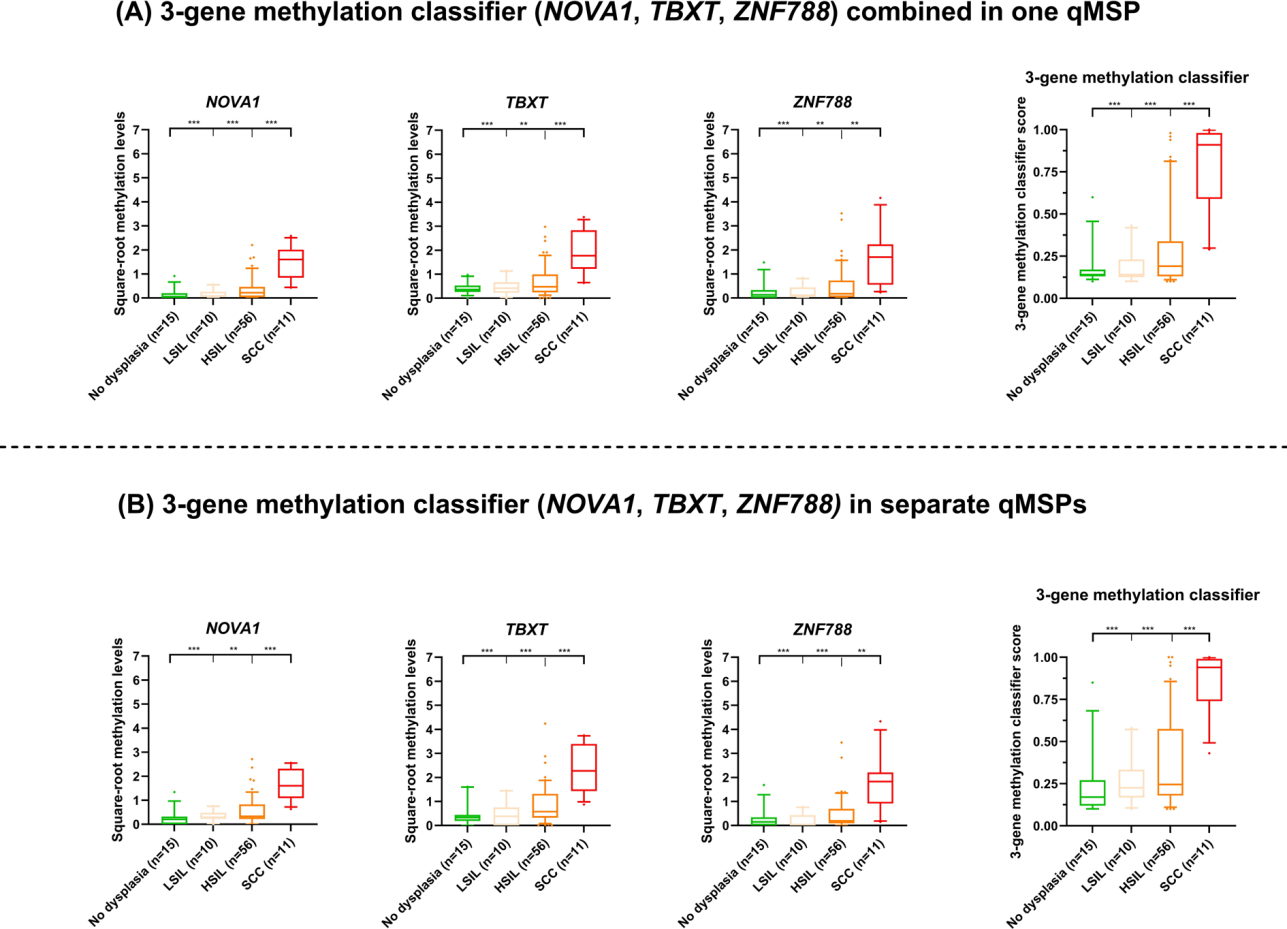
Comparison of methylation levels of genes *NOVA1*, *TBXT*, and *ZNF788* and the combined 3‐gene methylation classifier score using the 3‐gene classifier multiplex qMSP versus separate qMSP assays. DNA methylation levels, expressed as square‐root transformed ΔΔCq ratios, and the 3‐gene methylation classifier score are plotted against primary histological outcomes. Statistical differences between groups were evaluated using the Mann–Whitney *U* test with correction for multiple comparisons. Statistical differences are shown for SCC versus other histological categories and for HSIL versus other histological categories (if significant). Solid line represents the median, boxplot represents the 25th (Q1) and 75th (Q3) percentile. Whiskers range from the 10th to the 90th percentile. (A) Methylation levels of genes *NOVA1*, *TBXT*, and *ZNF788* and the combined 3‐gene methylation classifier score using the 3‐gene classifier multiplex qMSP. (B) Methylation levels of genes *NOVA1*, *TBXT*, and *ZNF788* and the combined 3‐gene methylation classifier score using separate qMSP assays. *p ≤ 0.05; **p ≤ 0.01; ***p ≤ 0.001; Abbreviations: HSIL, High‐grade squamous intraepithelial lesion; LSIL, Low‐grade squamous intraepithelial lesion; qMSP, Quantitative methylation‐specific PCR; SCC, Anal squamous cell carcinoma.

In secondary analyses, M^−^ HSIL and M^+^ HSIL were added as disease categories to classify HSIL lesions by their cancer risk according to the *ASCL1/ZNF582* methylation test on anal biopsies. Screening participants with M^+^ HSIL lesions tended to show higher methylation levels and classifier scores than those with M^−^ HSIL; however, these differences were not statistically significant after correction for multiple testing (*p* = 0.07 for *NOVA1, p* = 0.12 for *TBXT, p* = 0.63 for *ZNF788, p* = 0.04 for 3‐gene methylation classifier) (Figure S6).

Additionally, HPV16 infection was included as a disease category to classify HSIL lesions by their cancer risk. A similar pattern emerged, with HPV16^+^ HSIL lesions showing elevated methylation and classifier scores compared to HPV16^−^ lesions, although differences were not statistically different after multiple testing correction (*p* = 0.49 for *NOVA1, p* = 0.81 for *TBXT, p* = 0.61 for *ZNF788, p* = 0.35 for 3‐gene methylation classifier) (Figure S7). These findings were consistent with the performance of the individual markers and combined classifier when measured in separate multiplex assays on the same swab subset (Figure 4; Figures S6 and S7).

## 4 Discussion

Genome‐wide methylation profiling of anal swabs enabled the identification of a novel 3‐gene methylation classifier comprising *NOVA1*, *TBXT*, and *ZNF788* for the detection of HSIL:AIN3 and SCC in anal swab‐based screening. This classifier demonstrated excellent discriminatory performance in distinguishing HSIL:AIN3 and SCC from ≤LSIL lesions, achieving an AUC of 0.80 and successfully identifying all SCC cases. The inclusion of additional methylation markers did not significantly improve classifier performance.

Consistent with our previous findings [11, 19, 20, 21], methylation analysis of anal swabs from screening participants diagnosed with histological HSIL revealed a heterogeneous methylation pattern, with a subset exhibiting high methylation levels comparable to those observed in SCC swabs (i.e., cancer like methylation profile). To examine the relation between swab‐based methylation patterns and HSIL cancer risk, HSIL lesions were stratified by biopsy methylation status and classified as positive or negative according to the *ASCL1/ZNF582* methylation test. A positive test outcome served as a proxy for increased cancer risk, as demonstrated in our previous studies [19]. Screening participants with *ASCL1/ZNF582* methylation‐positive HSIL biopsies exhibited significantly higher methylation levels in paired swabs, both for our individual markers and 3‐gene methylation classifier, compared to those with *ASCL1/ZNF582* methylation‐negative biopsies. Similarly, anal swabs from participants with HSIL:AIN3 and HPV16^+^ HSIL lesions showed elevated methylation levels for both individual markers and 3‐gene methylation classifier. The 3‐gene methylation classifier accurately identified all SCC cases and was able to detect the vast majority of M^+^ HSIL and HPV16^+^ HSIL cases, supporting the utility of anal swab‐based methylation testing to stratify patients at higher cancer risk who may benefit from prioritized HRA referral or treatment. Given the use of surrogate endpoints in this study, further validation on swabs from confirmed regressive, nonregressive, and progressive HSIL lesions is warranted, for which specimens collected in the prospective MARINE and ANCHOR studies may be valuable [4, 38].

The newly developed 3‐gene methylation classifier *NOVA1/TBXT/ZNF788* demonstrated promising potential for several clinical applications. First, as a primary swab‐based screening tool, it may identify high‐risk individuals requiring referral or more frequent follow‐up. In our recent study comparing 20 screening algorithms, primary hrHPV testing detected over 85% of HSIL cases but missed 30% of anal cancers [10]. While the methylation classifier detects fewer HSIL overall, it identifies more HSIL lesions with a higher risk of progression to cancer, as well as all anal cancers. Compared to cytology, methylation analysis provides objective results and successfully detects all anal cancers [39, 40]. Second, the classifier could serve as a triage test to better stratify risk of progression to cancer among patients positive for hrHPV or cytology as methylation has been shown to be a promising prognostic marker for HSIL progression to cancer [19, 20]. Therefore, methylation testing has the potential to filter out lesions likely to regress, refining HRA referral. Cytology triage has been shown to improve specificity and reduce referrals in hrHPV‐positive MSMLWH [9], whereas the methylation classifier probably offers greater objectivity and superior discrimination between high‐ and low‐risk lesions. Third, the classifier may also serve as a triage tool for individuals who test positive for high‐risk HPV (hrHPV) types other than HPV16. In our cohort, 39 HSIL cases were positive for non‐HPV16 hrHPV types. Of these, over 60% (24/39) and nearly 50% (19/39) were methylation‐positive at thresholds of 0.20 and 0.25, respectively (data not shown). Moreover, among non‐HPV16 hrHPV‐positive HSIL patients with a methylation‐positive biopsy, 75% were also methylation‐positive on corresponding swab samples at both thresholds. Fourth, the methylation classifier may act as a quality control measure when HRA results are normal, as positive methylation correlates with severe HSIL and could warrant repeat examination. Fifth, a negative outcome of the methylation classifier may be useful for ruling out cancer, a feasibility previously suggested in cervical cancer research [41]. Further studies are required to establish the added value of incorporating the methylation classifier *NOVA1/TBXT/ZNF788* into anal swab‐based screening algorithms.

The *ASCL1/ZNF582* methylation test has demonstrated strong performance when applied to anal biopsies. All HSIL lesions that progressed to cancer in a longitudinal series, as well as all anal cancers, tested positive and an AUC of 0.81 (95% CI: 0.72–0.90) was observed for detecting histological HSIL:AIN3 and SCC [19]. When applied to anal swabs, the *ASCL1/ZNF582* methylation test showed a reduced AUC of 0.74 (95% CI: 0.66–0.83) for detecting histological HSIL:AIN3 and SCC [11], suggesting that the efficacy of methylation markers may be influenced by the sample type. Similarly, a recent multicenter study by Ferré et al. on patients with a history of AIN3 reported an AUC of 0.72 for both *ASCL1* (95% CI: 0.65–0.79) and *ZNF582* (95% CI: 0.66–0.79) for discriminating HSIL and non‐HSIL cytology by *ASCL1/ZNF582* methylation testing on anal swabs [42]. In the current study, evaluation of the newly developed swab‐derived 3‐gene methylation classifier *NOVA1/TBXT/ZNF788* on a screening population demonstrated improved diagnostic performance with an AUC of 0.80 (95% CI: 0.72–0.88). These findings suggest that the swab‐derived classifier might be better suited for anal swab‐based screening.

Supporting this, other studies assessing other biopsy‐ or cervical cancer–derived methylation markers on anal swabs have reported similarly lower performance. Phillips et al. evaluated *CADM1*, *MAL*, and *miR124‐2* methylation in anal swabs, observing only modest differences between HSIL and normal samples for *CADM1* and no significant differences for *MAL* and *miR124‐2* [15]. Similarly, Vasavada et al. examined the performance of *ASCL1* and *FMN2* in anal brush samples, reporting an AUC of 0.73 for distinguishing ≤LSIL from HSIL among people living with HIV, using histology and cytology as an endpoint [14]. Lahiri et al. evaluated *miR124‐2* and *FAM19A4* on anal swabs and found no difference in methylation levels between women with versus without HSIL [43]. Collectively, these findings suggest that the swab‐derived 3‐gene methylation classifier *NOVA1/TBXT/ZNF788* may offer stronger diagnostic potential than currently available methylation markers and marker combinations for anal swab‐based screening. A critical next step toward clinical implementation will be external validation and performance assessment in independent cohorts that include at‐risk populations other than MSMLWH. Additionally, head‐to‐head comparisons of the different tests on the same set of swabs are warranted.

Among the three genes in the 3‐gene methylation classifier, *NOVA1* contributed the most. *NOVA1* (Neuro‐Oncological Ventral Antigen 1) is a neuronal RNA‐binding protein that plays an important role in carcinogenesis by regulating cell growth, migration, apoptosis, and telomerase activity [44]. *NOVA1* is involved in Notch and Wnt signaling, which are also disrupted in HPV‐driven cancers [45, 46]. Furthermore, *NOVA1* methylation has prognostic potential in cervical cancer [47]. *TBXT* (T Brachyury Transcription Factor) is a driver of epithelial‐mesenchymal transition (EMT) [48], a critical process for tumor invasion and metastasis. Aberrant methylation and expression of *TBXT* have been linked to multiple carcinomas [48, 49]. Limited information is available about *ZNF788* (Zinc Finger Family Member 788) beyond its nucleic acid‐binding function, though its methylation appears to be associated with reduced kidney function [50]. Further research into the role of these genes, particularly *ZNF788*, in HPV‐driven anal carcinogenesis is warranted to determine the biological significance of their gene methylation.

A key strength of this study is that it is the first to develop a swab‐derived methylation classifier specifically for anal swabs. Although biopsy‐derived markers have demonstrated efficacy, we hypothesized that swab‐derived markers would be more suitable for anal swab samples. By conducting genome‐wide methylation profiling, we adopted an unbiased and comprehensive approach, allowing the inclusion of all potential markers. The identification of this classifier required the use of co‐data, which acts as a loop to help identify the “needle in the haystack”. Notably, all the top 50 candidate markers were also present within the co‐data, underscoring the relevance of this integrative approach in refining biomarker discovery.

Limitations of this study are that we only focused on MSMLWH due to the limited number of other at‐risk groups in our screening population. As a result, we cannot exclude the possibility that certain methylation markers may perform differently in individuals without HIV or women. Nonetheless, in a previous study, we demonstrated that other methylation markers tested in MSMLWH performed comparably in HIV‐negative individuals [24]. In our future validation study, we aim to include other risk groups, including HIV‐positive (trans)women and HIV‐negative individuals. Secondly, we implemented a quality control threshold excluding samples with more than 10% of CpG sites indistinguishable from background noise, in contrast to the commonly accepted threshold of 4%. This less stringent cut‐off was chosen to minimize sample loss and reduce exclusions from 15 to 9 out of 86 anal swabs. To assess the impact of this adjustment, we performed principal component analyses before and after applying the revised threshold. The results revealed comparable clustering patterns, principal components, and explained variance, indicating that the inclusion of these additional samples did not affect the overall data structure, thereby justifying supporting their retention in the analysis.

Through genome‐wide methylation analysis, we identified a swab‐derived 3‐gene methylation classifier consisting of *NOVA1*, *TBXT*, and *ZNF788*. This classifier demonstrated excellent discriminatory performance in distinguishing HSIL:AIN3 and SCC from ≤LSIL lesions, achieving an AUC of 0.80 and successfully identifying all SCC cases. The next critical step toward clinical implementation is the validation of this classifier in an independent external cohort of anal swabs, including populations at risk other than MSMLWH.

## Author Contributions

**Kirsten Rozemeijer:** methodology, writing – original draft, writing – review and editing, visualization, validation, data curation, formal analysis, investigation, project administration. **Jürgen Claesen:** methodology, formal analysis, investigation, writing – original draft, writing – review and editing, visualization. **Fernando Dias Gonçalves Lima:** resources, writing – review and editing. **Albertus T. Hesselink:** resources, writing – review and editing. **Phoebe Klingers:** resources, writing – review and editing. **Timo ter Braak:** resources, writing – review and editing. **Yara van den Burgt:** resources, writing – review and editing. **Chris J. L. M. Meijer:** conceptualization, writing – review and editing. **Jan M. Prins:** funding acquisition, conceptualization, resources, writing – review and editing. **Henry J. C. de Vries:** funding acquisition, conceptualization, resources, writing – review and editing. **Mark A. van de Wiel:** funding acquisition, conceptualization, methodology, formal analysis, investigation, writing – review and editing, supervision. **Renske D. M. Steenbergen:** funding acquisition, conceptualization, methodology, resources, writing – review and editing, supervision.

## Funding

The collaboration project was financed by the Ministry of Economic Affairs by means of the PPP Allowance made available by the Top Sector Life Sciences & Health to stimulate public‐private partnerships. Additional financial support was obtained by the Dutch Cancer Society (KWF17009) and the Stichting NEXTGEN HIGHTECH Program (Biomed02). Mark A. van de Wiel was financially supported by Stichting Hanarth Fonds.

## Ethics Statement

Ethical approval was waived by the Institutional Review Board of the Amsterdam UMC, location AMC, under reference numbers 18/316 (paired anal swabs and biopsies of AIN) and 18/333 (swabs of anal cancer).

## Consent

All participants gave written informed consent.

## Conflicts of Interest

K.R. received consultancy fees from FUJIREBIO paid to her institution. R.D.M.S. and C.J.L.M.M. are minority stockholders of Self‐screen B.V., a spin‐off company of VUmc, which owns patents for methylation markers and HPV detection. R.D.M.S. received consultancy fees from AstraZeneca paid to her institution. C.J.L.M.M. is part‐time CEO of Self‐screen B.V. and serves occasionally on the scientific advisory board of QIAGEN. A.T.H. and P.K. are employees of Self‐screen B.V. H.J.C.V. received financial compensation or goods for research from Medigene, Gilead, and MSD; financial compensation for presentations from Abbott and Janssen; and financial compensation for advice to Medigene and Novartis. All other authors declare no conflicts of interest. Amsterdam UMC has filed a patent on the results described in this manuscript.

## Supporting information


**Figure S1:** Overview of study populations before and after exclusion.
**Figure S2:** Overview of the probe selection.
**Figure S3:** Selection of candidate CpG sites by adaptive group‐regularized co‐data learnt ridge regression.
**Figure S4:** Methylation levels of the ten candidate markers in anal swabs, stratified by methylation status.
**Figure S5:** Methylation levels of the ten candidate markers in anal swabs, stratified by HPV16 status.
**Figure S6:** Comparison of methylation levels of genes *NOVA1*, *TBXT*, and *ZNF788* and the combined 3‐gene methylation classifier score using the 3‐gene classifier multiplex qMSP versus separate qMSP assays, stratified by methylation status.
**Figure S7:** Comparison of methylation levels of genes *NOVA1*, *TBXT*, and *ZNF788* and the combined 3‐gene methylation classifier score using the 3‐gene classifier multiplex qMSP versus separate qMSP assays, stratified by HPV16 status.

## Data Availability

The genome‐wide DNA methylation profiling dataset generated and analyzed in this study is available in the European Genome‐Phenome Archive repository, under accession number EGAD00010002832. Access of the profiling data can be requested by contacting the corresponding author. All source code is publicly available at https://doi.org/10.5281/zenodo.20038727. Further data that support the findings of this study are available from the corresponding author upon reasonable request.
