# Supplementary material for: Toward Objective Anal Cancer Screening: From Swab‐Based Genome‐Wide Methylation Marker Discovery to Novel Test Development
Source: Int J Cancer. 2026 Aug 27;160(1):187–200. doi: 10.1002/ijc.70708 (PMC13729930; doi:10.1002/ijc.70708)
Supplement: Supplementary file 1 [file IJC-160-187-s001.pdf]

Towards Objective Anal Cancer Screening: From Swab-Based Genome-Wide Methylation Marker Discovery to Novel Test Development

Kirsten Rozemeijer, Jürgen Claesen, Fernando Dias Gonçalves Lima, Albertus T. Hesselink, Phoebe Klingers, Timo ter Braak, Yara van den Burgt, Chris J.L.M. Meijer, Jan M. Prins, Henry J.C. de Vries, Mark A. van de Wiel, Renske D.M. Steenbergen

Table of content

Table of content	Page
Supplementary Figure 1	2
Supplementary Figure 2	3
Supplementary Figure 3	4
Supplementary Figure 4	5-6
Supplementary Figure 5	7-8
Supplementary Figure 6	9-10
Supplementary Figure 7	11-12
References	17

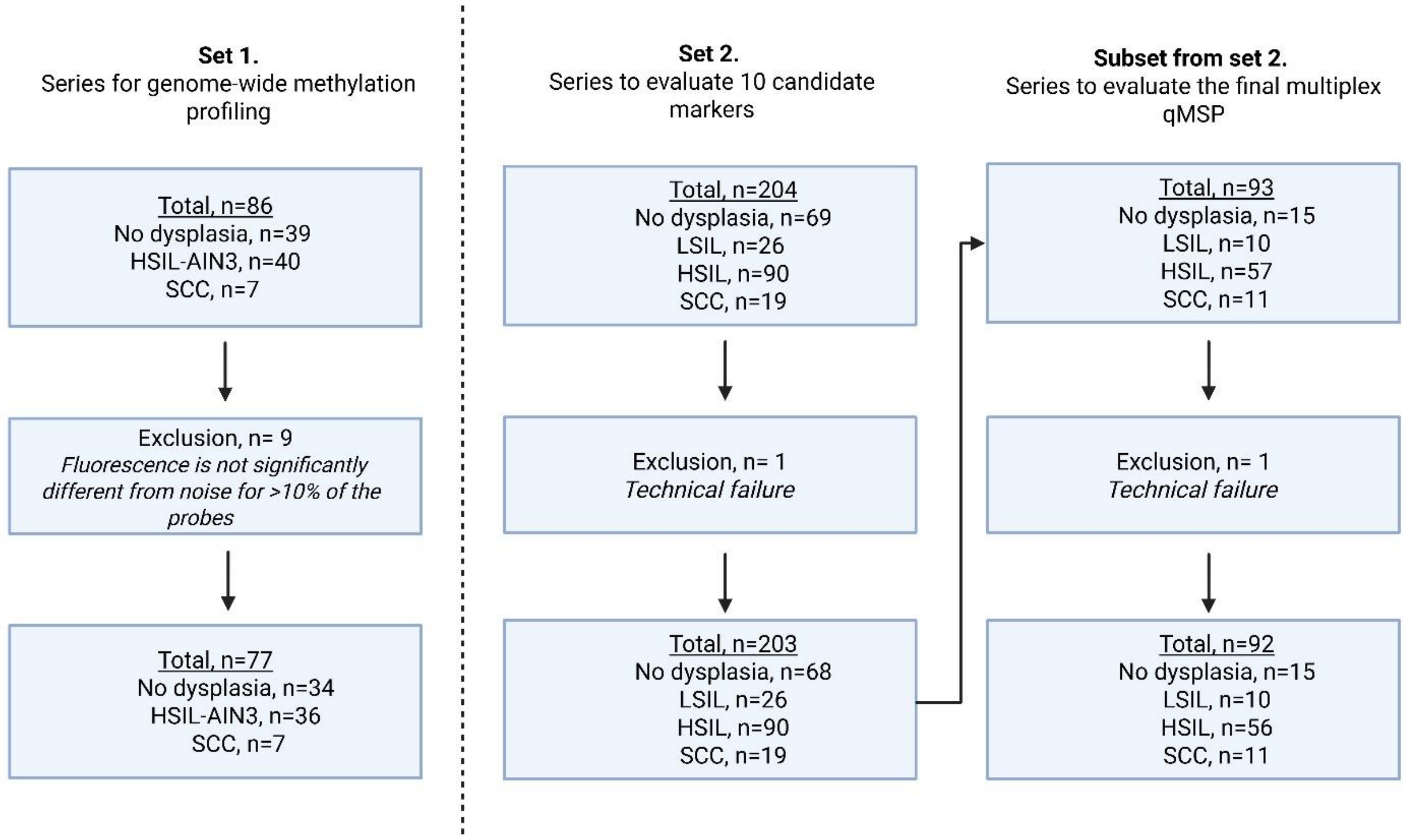

Supplementary Figure 1. Overview of study populations before and after exclusion.

Created with BioRender.com

Abbreviations: AIN: anal intraepithelial lesion; Ct: cycle threshold; HSIL: high-grade squamous intraepithelial lesions; LSIL: low-grade squamous intraepithelial lesion: qMSP: quantitative methylation specific PCR; SCC: anal squamous cell carcinoma; SCC: anal squamous cell carcinoma.

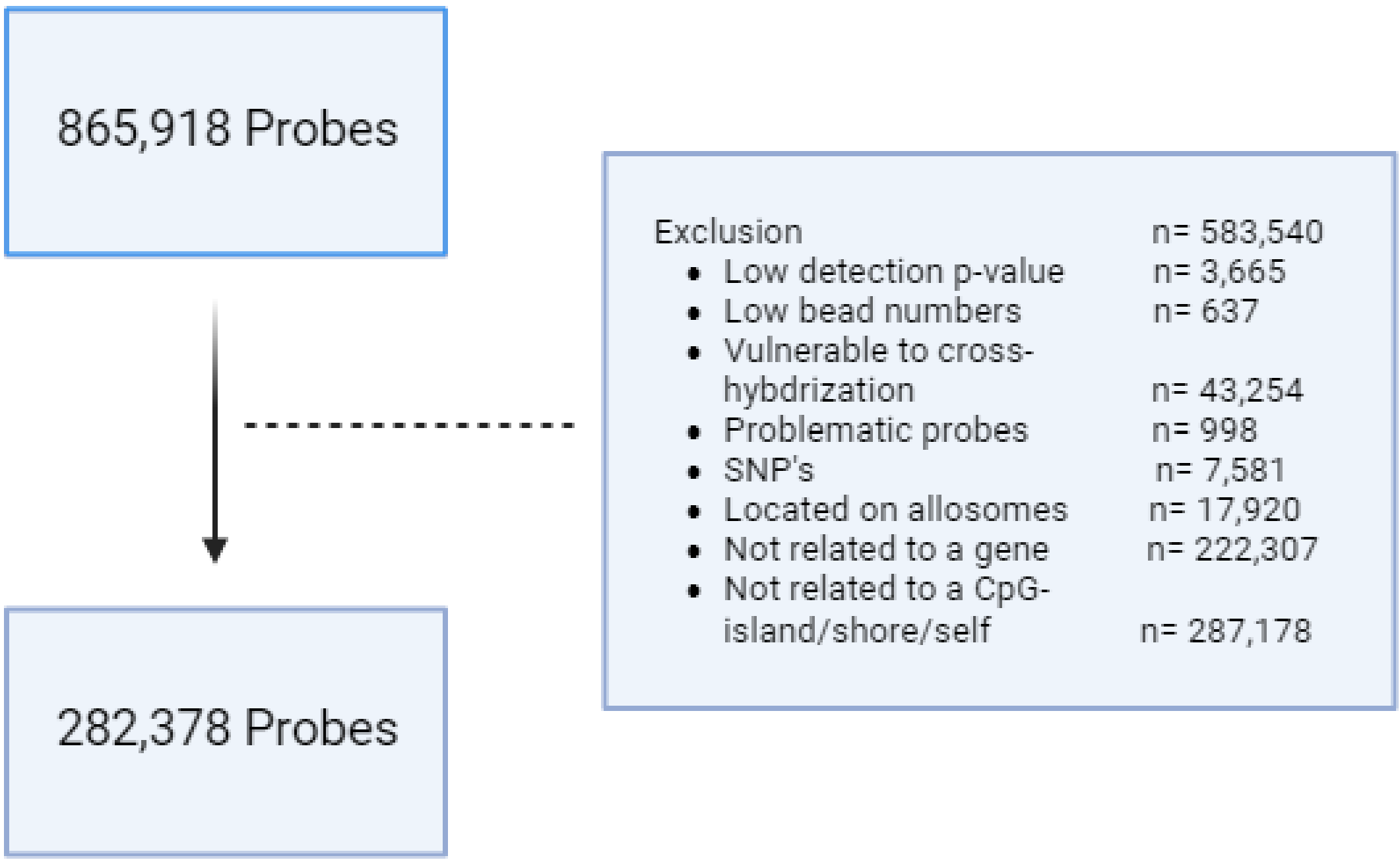

Supplementary Figure 2. Overview of the probe selection

Every probe corresponds to one CpG.

Created with BioRender.com

Abbreviations: SNP: single nucleotide polymorphism; CpG: cytosines followed by guanine residues.

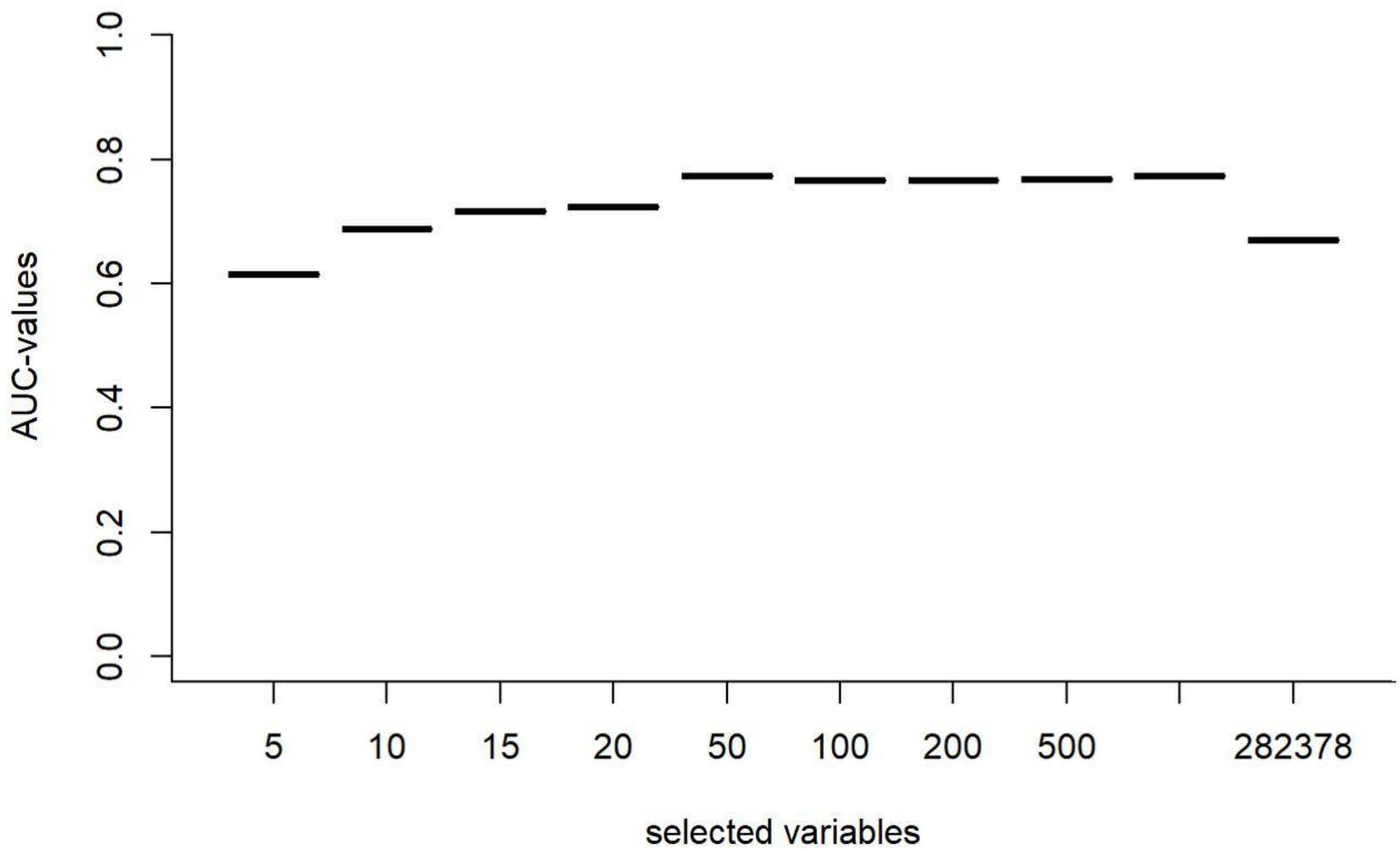

Supplementary Figure 3. Selection of candidate CpG sites by adaptive group-regularized co-data learnt ridge regression. The model containing 50 candidate CpG sites had the highest AUC of 0.80.

Abbreviations: AUC: area under the curve.

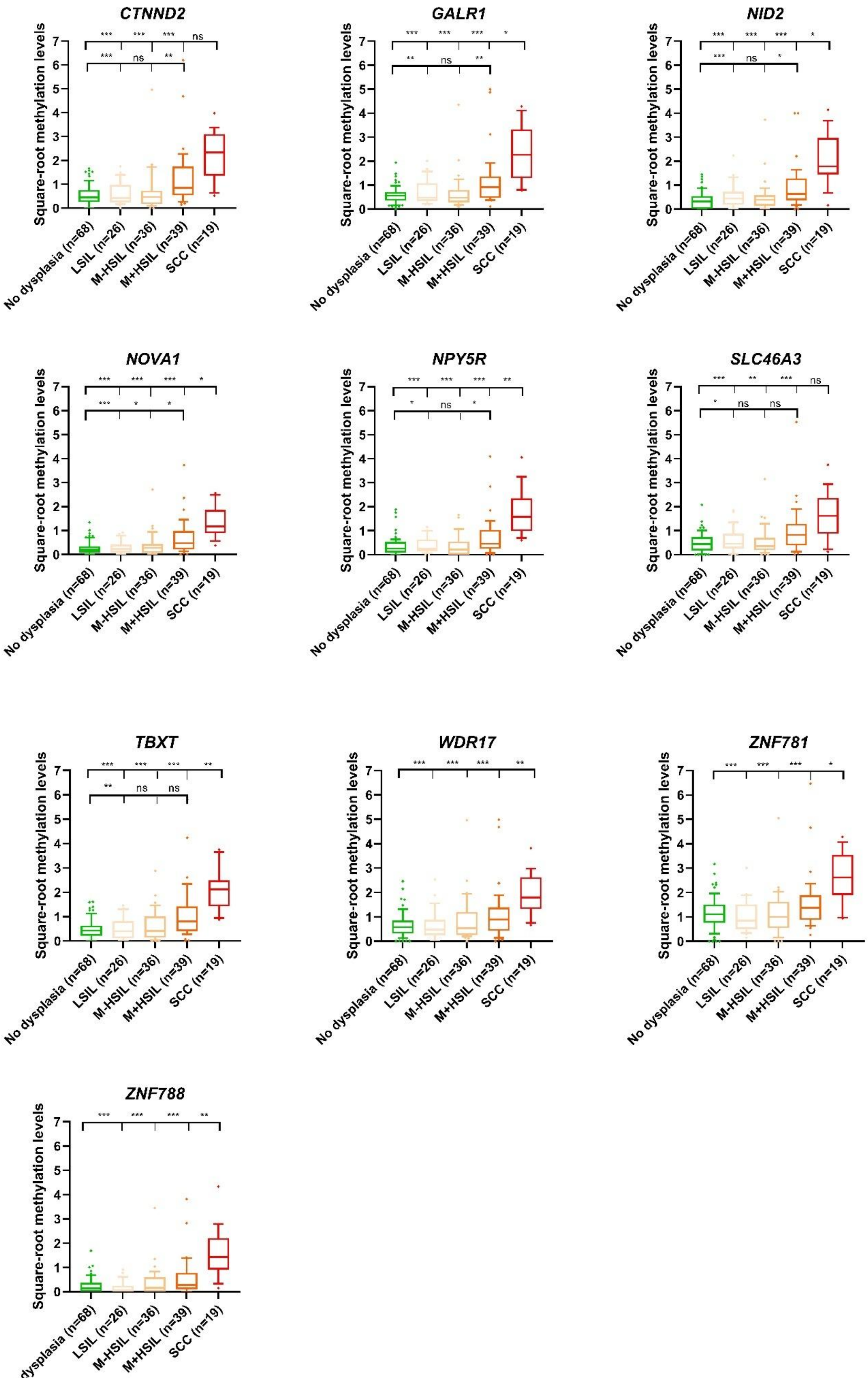
CTNND2
*** *** *** ns
*** ns **
GALR1
*** *** *** *
** ns **
NID2
*** *** *** *
*** ns *
NOVA1
*** *** *** *
*** * *
NPY5R
*** *** *** **
* ns *
SLC46A3
*** ** *** ns
* ns ns
TBXT
*** *** *** **
** ns ns
WDR17
*** *** *** **
ZNF781
*** *** *** *
ZNF788
*** *** *** **
Square-root methylation levels
0
1
2
3
4
5
6
7
No dysplasia (n=68)
LSIL (n=26)
M-HSIL (n=36)
M+HSIL (n=39)
SCC (n=19)

Supplementary Figure 4. Methylation levels of the ten candidate markers in anal swabs, stratified by methylation status. DNA methylation levels measured by qMSP and expressed as square-root transformed ΔΔCq ratios, are plotted against secondary histological outcomes. HSIL lesions are further classified based on biopsy methylation status – classified as positive or negative according to the *ASCL1/ZNF582* methylation test, a proxy for increased cancer risk as demonstrated in our previous studies(1, 2). HSIL with biopsies negative for methylation (M^- HSIL) represent low-risk lesions, while HSIL with biopsies positive for methylation (M^+ HSIL) represent high-risk lesions. Statistical differences between groups were evaluated using the Mann-Whitney *U* test with correction for multiple comparisons. Statistical differences are shown for SCC *versus* other histological categories and for M^+ HSIL *versus* other histological categories (if significant). Boxplots display the median (solid line), interquartile range (25th [Q1] to 75th [Q3] percentile), and whiskers indicate the 10th to 90th percentiles. Fifteen swabs from screening participants with HSIL were excluded due to unknown biopsy methylation status. For *NID2 and WDR17*, one datapoint is not shown (NID2: No dysplasia, 13.00; WDR17: No dysplasia, 7.84).

* = $p \leq 0.05$; ** = $p \leq 0.01$; *** = $p \leq 0.001$;

Abbreviations: HSIL: high-grade squamous intraepithelial lesion; LSIL: low-grade squamous intraepithelial lesion; M^- HSIL: HSIL biopsy negative for methylation (i.e., determined by the *ASCL1/ZNF582* methylation test); M^+ HSIL: HSIL biopsy positive for methylation (i.e., determined by the *ASCL1/ZNF582* methylation test); SCC: squamous cell carcinoma.

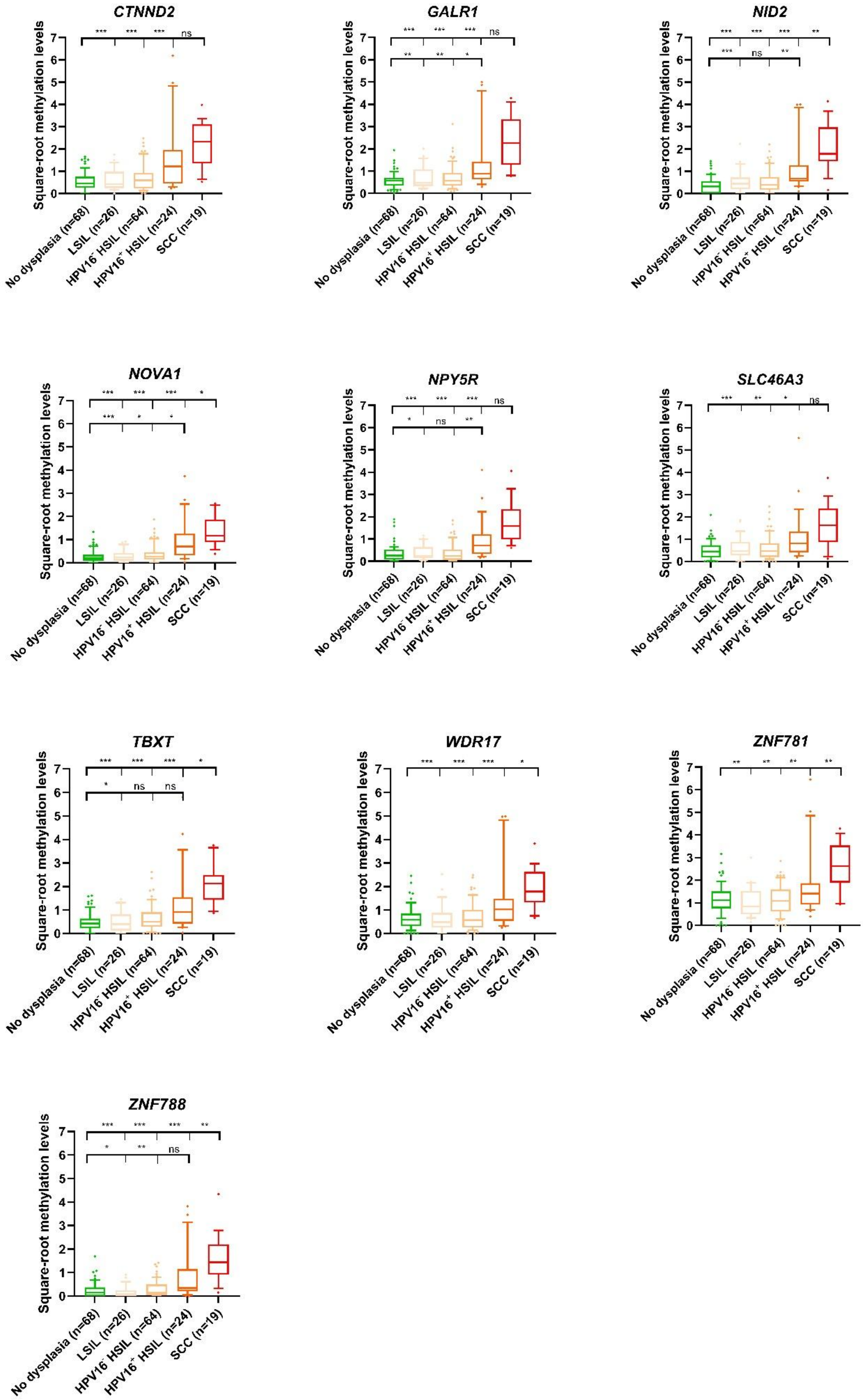
CTNND2
*** *** *** ns
GALR1
*** *** *** ns
** ** *
NID2
*** *** *** **
*** ns **
NOVA1
*** *** *** *
*** * *
NPY5R
*** *** *** ns
* ns **
SLC46A3
*** ** * ns
TBXT
*** *** *** *
* ns ns
WDR17
*** *** *** *
ZNF781
** ** ** **
ZNF788
*** *** *** **
* ** ns
Square-root methylation levels
No dysplasia (n=68)
LSIL (n=26)
HPV16⁻ HSIL (n=64)
HPV16⁺ HSIL (n=24)
SCC (n=19)

Supplementary Figure 5. Methylation levels of the ten candidate markers in anal swabs, stratified by HPV16 status. DNA methylation levels measured by qMSP and expressed as square-root transformed ΔΔCq ratios, are plotted against secondary histological outcomes. HSIL lesions are classified by HPV16 infection status: HPV16-negative HSIL ($HPV16^-$ HSIL) represent low-risk lesions, while HPV16-positive HSIL ($HPV16^+$ HSIL) represent high-risk lesions. Statistical differences between groups were evaluated using the Mann-Whitney *U* test with correction for multiple comparisons. Statistical differences are shown for SCC *versus* other histological categories and for $HPV16^+$ HSIL *versus* other histological categories (if significant). Boxplots display the median (solid line), interquartile range (25th [Q1] to 75th [Q3] percentile), and whiskers indicate the 10th to 90th percentiles. Two swabs were excluded due to unknown HPV16 status. For *NID2 and WDR17*, one datapoint is not shown (NID2: No dysplasia, 13.00; WDR17: No dysplasia, 7.84).

* = $p \leq 0.05$; ** = $p \leq 0.01$; *** = $p \leq 0.001$;

Abbreviations: $HPV16^-$ HSIL: Screening participant with a HSIL diagnoses and no HPV16 infection (as determined by hrHPV testing of the anal swab); $HPV16^+$ HSIL: Screening participant with a HSIL diagnoses and HPV16 infection (as determined by hrHPV testing of the anal swab); HSIL: high-grade squamous intraepithelial lesion; LSIL: low-grade squamous intraepithelial lesion; SCC: squamous cell carcinoma.

A) 3-gene methylation classifier (*NOVA1*, *TBXT*, *ZNF788*) combined in one qMSP

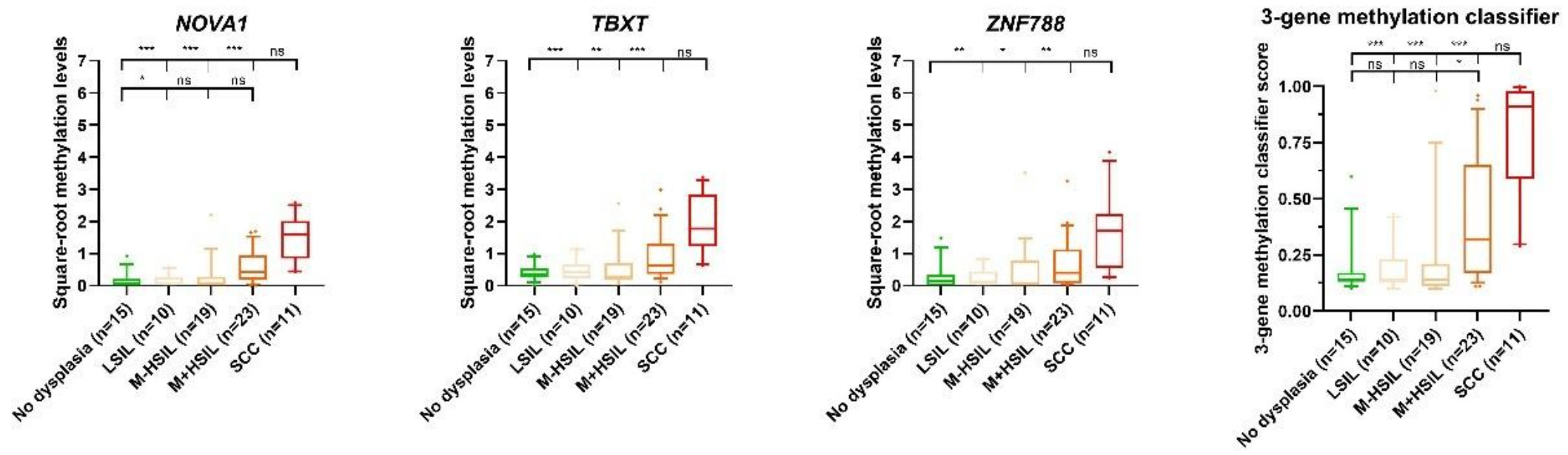

B) 3-gene methylation classifier (*NOVA1*, *TBXT*, *ZNF788*) in separate qMSP's

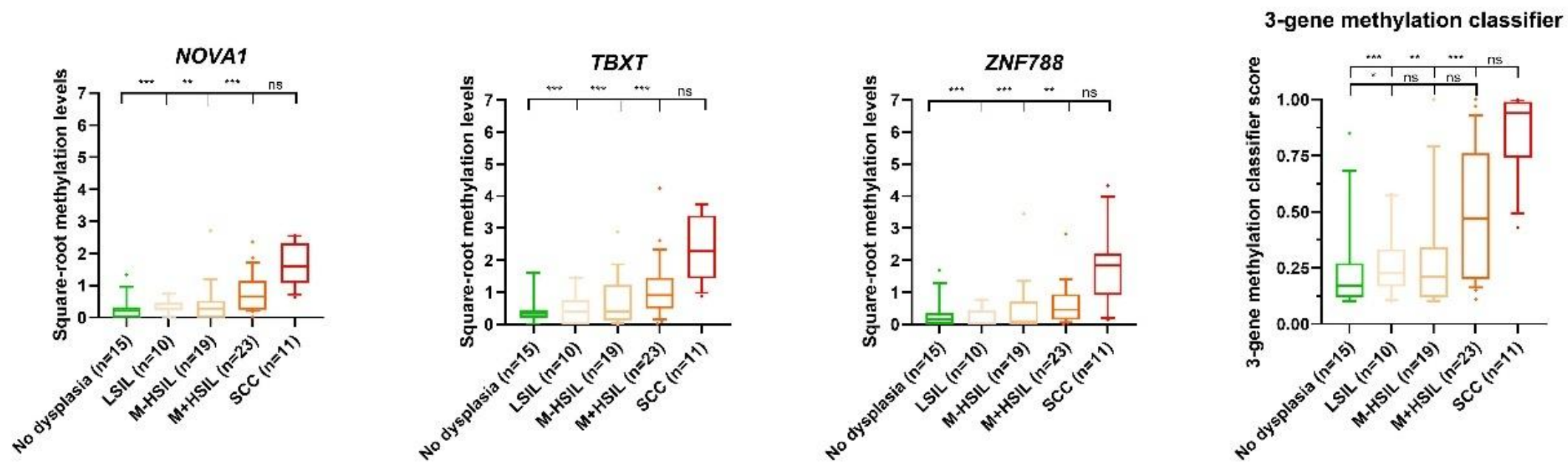

Supplementary Figure 6. Comparison of methylation levels of genes *NOVA1*, *TBXT*, and *ZNF788* and the combined 3-gene methylation classifier score using the 3-gene classifier multiplex qMSP *versus* separate qMSP assays, stratified by methylation status.

DNA methylation levels measured by qMSP and expressed as square-root transformed ΔΔCq ratios, and the 3-gene methylation classifier score are plotted against secondary histological outcomes. HSIL lesions are further classified based on biopsy methylation status – classified as positive or negative according to the *ASCL1/ZNF582* methylation test, a proxy for increased cancer risk as demonstrated in our previous studies(1, 2). HSIL with biopsies negative for methylation (M^- HSIL) represent low-risk lesions, while HSIL with biopsies positive for methylation (M^+ HSIL) represent high-risk lesions. Statistical differences between groups were evaluated using the Mann-Whitney *U* test with correction for multiple comparisons. Statistical differences are shown for SCC *versus* other histological categories and for M^+ HSIL *versus* other histological categories (if significant). Solid line represents the median, boxplot represents the 25th (Q1) and 75th (Q3) percentile. Whiskers range from the 10th to the 90th percentile.

A) Methylation levels of genes *NOVA1*, *TBXT*, and *ZNF788* and the combined 3-gene methylation classifier score using the 3-gene classifier multiplex qMSP.

B) Methylation levels of genes *NOVA1*, *TBXT*, and *ZNF788* and the combined 3-gene methylation classifier score using separate qMSP assays.

* = $p \leq 0.05$; ** = $p \leq 0.01$; *** = $p \leq 0.001$;

Abbreviations: HSIL: high-grade squamous intraepithelial lesion; LSIL: low-grade squamous intraepithelial lesion; M^- HSIL: HSIL biopsy negative for methylation (i.e., determined by the *ASCL1/ZNF582* methylation test); M^+ HSIL: HSIL biopsy positive for methylation (i.e., determined by the *ASCL1/ZNF582* methylation test); qMSP: quantitative methylation-specific PCR; SCC: squamous cell carcinoma.

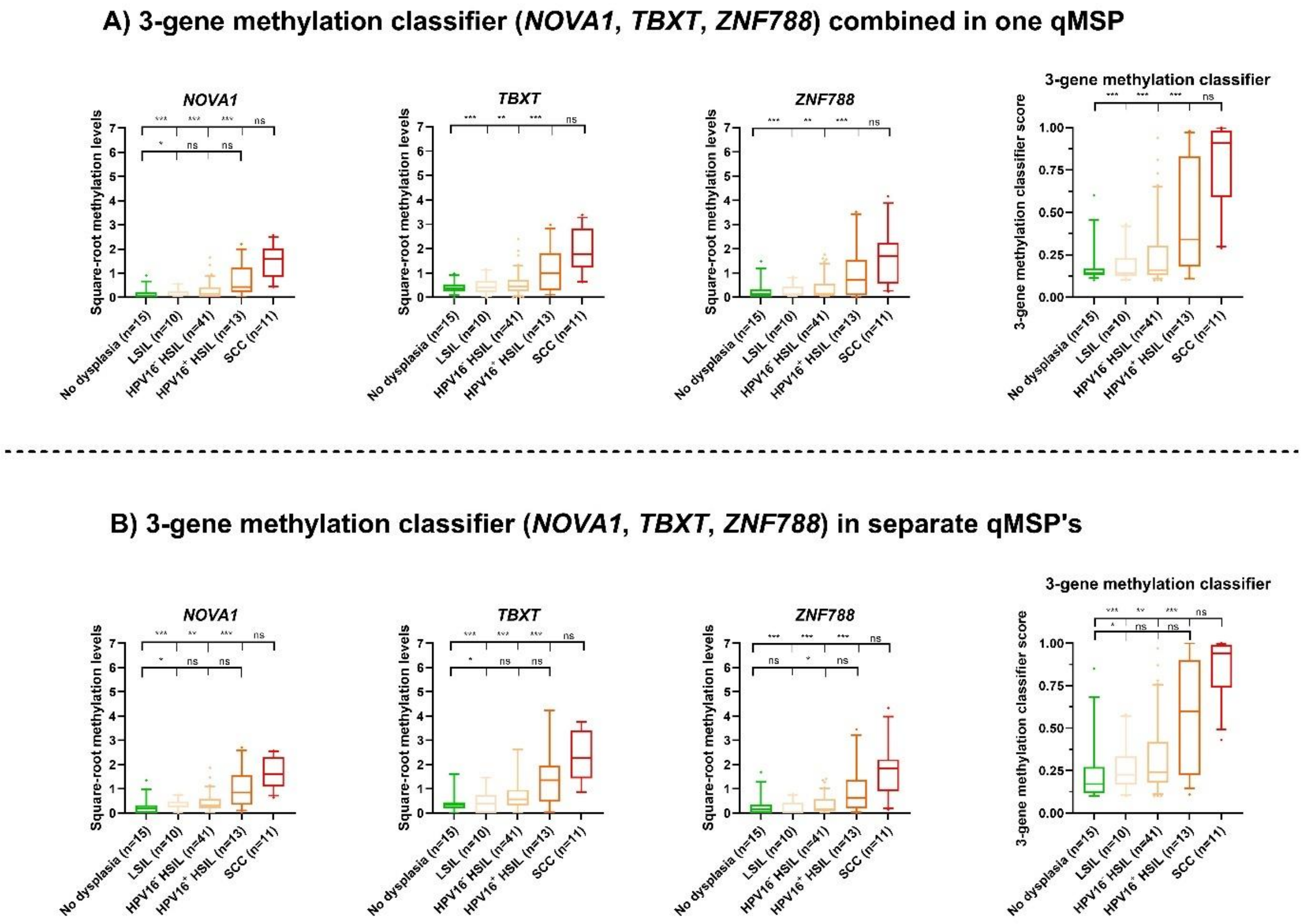

Supplementary Figure 7. Comparison of methylation levels of genes *NOVA1*, *TBXT*, and *ZNF788* and the combined 3-gene methylation classifier score using the 3-gene classifier multiplex qMSP *versus* separate qMSP assays, stratified by HPV16 status.

DNA methylation levels measured by qMSP and expressed as square-root transformed ΔΔCq ratios, and the 3-gene methylation classifier score are plotted against secondary histological outcomes. HSIL lesions are classified by HPV16 infection status: HPV16-negative HSIL ($HPV16^-$ HSIL) represent low-risk lesions, while HPV16-positive HSIL ($HPV16^+$ HSIL) represent high-risk lesions. Statistical differences between groups were evaluated using the Mann-Whitney *U* test with correction for multiple comparisons. Statistical differences are shown for SCC *versus* other histological categories and for $HPV16^+$ HSIL *versus* other histological categories (if significant). Solid line represents the median, boxplot represents the 25th (Q1) and 75th (Q3) percentile. Whiskers range from the 10th to the 90th percentile.

A) Methylation levels of genes *NOVA1*, *TBXT*, and *ZNF788* and the combined 3-gene methylation classifier score using the 3-gene classifier multiplex qMSP.

B) Methylation levels of genes *NOVA1*, *TBXT*, and *ZNF788* and the combined 3-gene methylation classifier score

using separate qMSP assays.

* = $p \leq 0.05$; ** = $p \leq 0.01$; *** = $p \leq 0.001$;

Abbreviations: $HPV16^{-}$ HSIL: Screening participant with a HSIL diagnoses and no HPV16 infection (as determined by hrHPV testing of the anal swab); $HPV16^{+}$ HSIL: Screening participant with a HSIL diagnoses and HPV16 infection (as determined by hrHPV testing of the anal swab); HSIL: high-grade squamous intraepithelial lesion; LSIL: low-grade squamous intraepithelial lesion; qMSP: quantitative methylation-specific PCR; SCC: squamous cell carcinoma.

References

1. Rozemeijer K, Dias Goncalves Lima F, Ter Braak TJ, Hesselink AT, Prins JM, de Vries HJC, et al. Analytical validation and diagnostic performance of the ASCL1/ZNF582 methylation test for detection of high-grade anal intraepithelial neoplasia and anal cancer. Tumour Virus Res. 2024;17:200275.
2. van der Zee RP, Richel O, van Noesel CJM, Ciocanea-Teodorescu I, van Splunter AP, Ter Braak TJ, et al. Cancer Risk Stratification of Anal Intraepithelial Neoplasia in Human Immunodeficiency Virus-Positive Men by Validated Methylation Markers Associated With Progression to Cancer. Clin Infect Dis. 2021;72(12):2154–63.
